# Light-Enhanced Electrochemical Performance of Fish Waste-Derived Carbon-TiO_2_ Composites for Sustainable Energy Storage Systems

**DOI:** 10.3390/nano16090538

**Published:** 2026-04-29

**Authors:** Ana T. S. C. Brandão, Sabrina State, Laura Bianca Enache, Renata Costa, Geanina Valentina Mihai, José A. Vázquez, Jesus Valcarcel, Liana Anicai, Marius Enachescu, Carlos M. Pereira

**Affiliations:** 1 Centro de Investigação em Química da Universidade do Porto/Instituto de Ciências Moleculares (CIQUP/IMS), Faculdade de Ciências da Universidade do Porto, Departamento de Química e Bioquímica, Rua do Campo Alegre 687, 4169-007 Porto, Portugal; renata.costa@fc.up.pt (R.C.); cmpereir@fc.up.pt (C.M.P.); 2 Faculty of Medical Engineering, National University of Science and Technology Politehnica Bucharest, 1-7 Gheorghe Polizu Street, 011061 Bucharest, Romania; sabrina.rosoiu@upb.ro; 3 National Institute for Research and Development in Microtechnologies-IMT Bucharest, 126A Erou Iancu Nicolae, 077190 Bucharest, Romania; 4 Center for Surface Science and Nanotechnology, National University of Science and Technology Politehnica Bucharest, Splaiul Independentei, 313, 060042 Bucharest, Romania; a.bianca@cssnt-upb.ro (L.B.E.); geanina.mihai@cssnt-upb.ro (G.V.M.); liana.anicai@cssnt-upb.ro (L.A.); marius.enachescu@cssnt-upb.ro (M.E.); 5 Grupo de Reciclado y Valorización de Materiales Residuales (REVAL), Instituto de Investigaciones Marinas (IIM-CSIC), 36208 Vigo, Spain; jvazquez@iim.csic.es (J.A.V.); jvalcarcel@iim.csic.es (J.V.); 6 Academy of Romanian Scientists, Splaiul Independentei 54, 050094 Bucharest, Romania

**Keywords:** fish waste, biocarbons, electrochemistry, titanium dioxide, light effect, capacitance

## Abstract

This work reports on the synthesis and electrochemical investigation of sustainable carbon–TiO_2_ nanocomposites derived from marine biowaste, designed to elucidate light-assisted charge storage mechanisms in non-aqueous electrolytes. Porous carbons obtained from prawn chitin and blue shark gelatin were decorated in situ with TiO_2_ nanoparticles using a deep eutectic solvent (DES) as a green synthesis medium. Structural and morphological characterisation revealed that TiO_2_ incorporation induces significant nanoscale reorganisation of the carbon framework, resulting in hierarchical porosity, increased surface area, and intimate semiconductor–carbon interfaces. Electrochemical evaluation in a three-electrode configuration using an ethaline-based DES electrolyte demonstrated that TiO_2_ decoration substantially enhances capacitive performance and cycling stability, with the prawn chitin-derived composite achieving a specific capacitance of 54 ± 3 F g^−1^ and 91% retention after 10,000 cycles. Under illumination, all TiO_2_-containing composites exhibited a pronounced increase in anodic current response and discharge time, indicating photo-assisted surface charge accumulation. Although the absolute capacitance values are modest compared to those of aqueous supercapacitor systems, the results provide mechanistic insight into the interplay among nanostructure, semiconductor photoactivity, and ion transport in viscous, hydrogen-bonded DES electrolytes. By combining waste-derived carbons, green synthesis routes, and photo-responsive nanostructures, this study highlights a sustainable strategy for developing multifunctional carbon-based nanomaterials with light-modulated electrochemical behaviour.

## 1. Introduction

The increasing need for high-performance, sustainable energy storage solutions that reduce reliance on scarce critical materials has driven the exploration of innovative materials for supercapacitor applications. These innovations are essential in supercapacitor applications since they are distinguished by their rapid charge/discharge rates, high power density, and extended cycle life, making them ideal for various applications (e.g., mobility electrification and portable electronics). A critical aspect of supercapacitor development is the selection of electrode materials that offer superior electrochemical performance while being environmentally sustainable [1,2].

Carbon materials are the cornerstone of supercapacitor electrodes due to their excellent electrical conductivity, large specific surface area, and chemical stability. Traditionally sourced from non-renewable resources (e.g., coal, petroleum), these materials have raised environmental concerns, leading to increased interest in renewable, waste-derived carbon sources [3]. As a result, exploring marine biowastes as alternatives addresses environmental concerns and capitalises on the potential of these abundant, underutilised resources to produce high-performance carbon materials for energy storage applications. Marine biowastes, such as prawn chitin [4] and blue shark gelatin [5], have emerged as promising precursors for the sustainable production of carbon materials. These biowastes contribute to waste reduction and offer unique structural properties that enhance their functionality in energy storage devices [4]. When carbonised, these precursors yield carbon materials with a high degree of porosity and abundant oxygen-containing functional groups [5]. These features are particularly advantageous for supercapacitor applications, as they enhance the material’s capacity for efficient energy storage and release [6]. Prawn chitin, derived from the exoskeletons of crustaceans, is composed of N-acetylglucosamine units, which, upon carbonisation, produce nitrogen-doped carbon [7]. This material is known for its porous structure, which facilitates ion transport and enhances charge storage. Similarly, blue shark gelatin, rich in amino acids, carbonises into a nitrogen-doped carbon with hierarchical porosity, offering significant surface functionalisation that further enhances electrochemical performance [8,9].

The surface area and specific capacitance of carbon materials derived from prawn chitin and blue shark gelatin, carbonised at 1000 °C for 1 h, exhibit significant variations based on the precursor’s origin and structural properties. Prawn chitin-based carbons, with a surface area of 85 ± 1 m^2^ g^−1^, showed a specific capacitance of 15 ± 2 F g^−1^ [10]. In contrast, the blue shark gelatin-derived carbon, with a surface area of 30 ± 1 m^2^ g^−1^, presented a lower capacitance of 7 ± 1 F g^−1^ [11]. Increasing the surface area of carbon materials is crucial for enhancing their performance as supercapacitor electrodes. A higher surface area provides more active sites for charge accumulation, allowing more electrolyte ions to interact with the electrode material. This interaction directly affects the material’s capacitance, thereby increasing its energy storage capacity. Furthermore, the porous structure of high-surface-area materials facilitates ion diffusion, improving supercapacitors’ efficiency and the rate of charge/discharge cycles. Thus, optimising surface area is key to developing high-performance energy storage devices.

An essential advancement in developing these carbon materials, increasing their surface area and conductivity, is their decoration with titanium dioxide (TiO_2_). TiO_2_ is a semiconductor well-known for its high surface reactivity, excellent chemical stability, and ability to enhance the capacitance and cycling stability of carbon-based electrodes [12]. The method of TiO_2_ production and its attachment to the carbon matrix are critical factors in determining the composite material’s performance. In this study, the decoration of prawn chitin and blue shark gelatin-derived carbon materials with TiO_2_ was achieved via an in situ synthesis method involving the anodisation of a titanium plate using a deep eutectic solvent (DES) as the electrolyte. This method was first described by Anicai et al. [13] for synthesising TiO_2_ nano powders and later adapted by Brandão et al. [14] for the decoration of biomass-based carbons. This innovation leveraged the unique properties of DES and an analogous ionic liquid class to enhance the synthesis process. DESs are formed by mixing a hydrogen bond acceptor, typically a quaternary ammonium salt, with a hydrogen bond donor such as ethylene glycol, urea, or glycerol [15], providing a green and versatile medium for nanomaterial synthesis. DESs are also considered green solvents, offering an environmentally friendly alternative to traditional solvents [16].

The use of DES for the in situ synthesis of TiO_2_ enables a uniform, controlled distribution of TiO_2_ nanoparticles on the surface of carbon materials. This method ensures strong interactions between TiO_2_ and the carbon matrix, enhancing the composite’s electrochemical properties. The TiO_2_-decorated carbon composites produced via this method exhibit improved surface area, pore volume, and charge storage capacity, making them highly suitable for supercapacitor applications [17].

Despite the extensive use of TiO_2_–carbon composites in electrochemical energy storage, most reported systems focus primarily on maximising capacitance values in conventional aqueous or organic electrolytes, often overlooking the mechanistic origins of charge storage and the role of non-traditional electrolytes. In particular, the contribution of light to charge storage in TiO_2_-containing supercapacitors remains insufficiently understood, especially in non-aqueous ionic environments where classical Ti^4+^/Ti^3+^ bulk redox processes are thermodynamically suppressed.

In recent years, photo-assisted electrochemical energy storage systems have attracted growing attention as a route to combine light harvesting with charge storage in a single functional platform. Recent reviews [18,19] show that this field now includes several overlapping but distinct approaches, such as integrated photovoltaic–supercapacitors, photoresponsive pseudocapacitive electrodes, and hybrid devices that couple solar energy conversion with capacitive storage. Within this broader landscape, TiO_2_ remains one of the most widely studied photoactive materials because of its stability, low cost, and well-established semiconductor behaviour. At the same time, carbonaceous components are frequently incorporated to enhance conductivity, interfacial charge separation, and charge-transport kinetics. Recent state-of-the-art analyses further show that TiO_2_-carbon systems are increasingly being designed not only for improved capacitance, but also for light-responsive and multifunctional operation. However, much of the recent literature has focused either on integrated device architectures or on highly optimised TiO_2_–graphene and polymer-containing systems, often tested in aqueous or device-specific electrolytes. By contrast, fewer studies have examined how precursor chemistry, TiO_2_ incorporation, and illumination jointly influence charge storage in sustainable biowaste-derived carbon electrodes operating in a DES environment.

DESs represent a distinct electrochemical medium characterised by high viscosity, asymmetric ion size, and strong hydrogen bond networks, which fundamentally alter ion transport and interfacial charge storage compared to aqueous systems. While DESs have been explored as green electrolytes, their interaction with semiconductor–carbon composites, particularly under illumination, remains largely unexplored.

In this context, the present work does not aim to establish record-level capacitance values, but rather to elucidate how TiO_2_ decoration and light exposure modulate charge storage in biowaste-derived carbons operating in a DES electrolyte. By combining marine waste-derived carbons with TiO_2_ synthesised and deposited in situ within a DES medium, this study highlights the structural, interfacial, and photo-assisted effects governing charge accumulation in these hybrid systems. The results provide mechanistic insight into light-enhanced capacitive behaviour under non-aqueous conditions while emphasising the sustainability and circular-economy aspects of the material design. Although no dedicated theoretical modelling is included in the present study, a mechanistic interpretation is developed by integrating structural, compositional, and electrochemical results to propose a consistent picture of light-assisted interfacial charge storage in the TiO_2_@C composites.

Despite the extensive use of TiO_2_–carbon composites in electrochemical energy storage, the novelty of the present work does not lie in the generic combination of biomass-derived carbon and TiO_2_, as such systems have been widely reported. Rather, this study distinguishes itself through the combined use of two chemically distinct marine waste precursors, prawn chitin and blue shark gelatin, the in situ synthesis and deposition of TiO_2_ within a DES medium, and the investigation of light-assisted charge storage behaviour in a non-aqueous DES electrolyte. This design enables a direct comparison of how different marine biopolymer chemistries influence the structure, porosity, interfacial properties, and electrochemical response of TiO_2_@C composites under dark and illuminated conditions. In this context, the present work does not aim to establish record-level capacitance values, but rather to elucidate how TiO_2_ decoration and light exposure modulate charge storage in biowaste-derived carbons operating in a DES electrolyte. By combining marine-waste-derived carbons with TiO_2_ synthesised and deposited in situ within a DES medium, this study highlights the structural, interfacial, and photo-assisted effects governing charge accumulation in these hybrid systems, while emphasising sustainability, circular-economy valorisation, and mechanistic insight under non-aqueous conditions.

The present study employs a three-electrode configuration, which imposes different transport conditions due to higher viscosity and structured ionic interactions. As a result, the capacitance values reported here are not directly comparable to those obtained in aqueous systems. Instead, this work focuses on understanding the interplay among material structure, semiconductor photoactivity, and ion transport under non-aqueous conditions. Therefore, the main contribution of this study lies not in maximising capacitance values, but in demonstrating a sustainable marine-waste-derived TiO_2_–carbon system with light-assisted charge-storage behaviour in a DES electrolyte, thereby providing insight into electrochemical performance under non-traditional conditions.

## 2. Materials and Methods

### 2.1. Biocarbon Derived from Fish Waste Preparation

Gelatine was recovered from the discarded skins of blue sharks [20], and chitins were recovered from prawn shells [21]. The raw biomass was placed in a tubular furnace at 1000 °C for 1 h under a 0.3 L h^−1^ N_2_ flow-controlled environment to collect the ashes (45 wt.% recovery). The samples obtained after carbonisation will be abbreviated as PC (prawn chitin) and BSG (blue shark gelatine).

### 2.2. Preparation of Deep Eutectic Solvent (DES)

The eutectic mixture was prepared according to the method previously described by Brandão et al. [22]. Briefly, choline chloride (ChCl, Sigma-Aldrich, 99%, St. Louis, MO, USA) was mixed with ethylene glycol (Sigma-Aldrich, 99%, MO, USA) in a 1:2 molar ratio and heated to 60 °C. The mixture was stirred thoroughly under controlled conditions until it reached a clear, homogeneous liquid phase, forming a DES commonly designated ethaline. This transparent and uniform solution, composed of choline chloride as a hydrogen-bond acceptor and ethylene glycol as a hydrogen-bond donor, is crucial for facilitating subsequent synthesis steps by providing a stable, efficient medium for in situ decoration of carbon materials. The water content of the resulting deep eutectic solvent (DES) was measured immediately after preparation at room temperature using a Karl Fischer titrator (831 KF Coulometer, Methrom, Herisau, Switzerland), yielding a value of 7.6 ± 0.3 wt.% [23].

To compare the performance of the DES electrolytes with that of aqueous and organic electrolytes the best performing material (PC) was tested with two aqueous electrolytes (1 mol dm^−3^ sulfuric acid (H_2_SO_4_, Sigma-Aldrich, 99%, MO, USA) and 6 mol dm^−3^ potassium hydroxide (KOH, Sigma-Aldrich, 99%, MO, USA)) and one organic electrolyte (1 mol dm^−3^ triethylmethylammonium tetrafluoroborate (NEt_3_MeBF_4_, Sigma-Aldrich, 99%, MO, USA) in a 60:40 (v:v) mixture of dimethyl carbonate (DMC, Sigma-Aldrich, 99%, MO, USA), ethyl methyl carbonate (EMC, Sigma-Aldrich, 99%, MO, USA), respectively).

### 2.3. Electrochemical Synthesis of TiO_2_ Nanopowders Through the Anodic Dissolution of a Ti Metal

The electrochemical synthesis of TiO_2_ was carried out using the method described by Anicai et al. [13]. The experiments were performed under stationary conditions using an open system powered by a DC power supply (0–5 A, 0–30 V, AX-3005DS AXIOMET). The electrochemical cell featured a two-electrode setup containing 200 mL of a choline chloride-ethylene glycol eutectic mixture as the electrolyte, along with 100 mL of ethanol and 5 mmol L^−1^ (total volume) of tetrabutylammonium bromide (TBAB). A pure titanium disc (4 mm thick) with an exposed area of 38.46 cm^2^ was used as the sacrificial anode, while nickel strips (0.3 mm thick, 13.5 cm^2^) served as the cathode. To attach TiO_2_ to the carbon matrix, 0.3 g of biocarbon was added to the electrolyte and maintained in suspension during TiO_2_ formation. This procedure was already described by Brandão et al. [14].

The anodic and cathodic areas were kept at a 1:1 ratio. A 40 mA cm^−2^ current density was applied for 3 h under constant mechanical stirring at a controlled temperature of 45 °C. Following dissolution of the Ti anode (terminated after 3 h), 10 mL of ultrapure water was added to the cell electrolyte, resulting in direct hydrolysis and the formation of a white gel. This white gel, containing TiO_2_ nanoparticles, was then cleaned with hot water and ethanol, followed by centrifugation at 4000 rpm for 15 min. This process was repeated four times, and the final product was dried at 110 °C for 1 h. After TiO_2_ is attached to the carbon matrix, the resulting composite samples will be named PC+TiO_2_ and BSG+TiO_2_. Figure S1 presents the carbon material dispersed in the eutectic mixture during the formation of the TiO_2_ nano powders (a) and the biocarbon-TiO_2_ composite after washing.

Due to the in situ electrochemical formation of TiO_2_ via anodic dissolution of the Ti electrode, the exact TiO_2_ loading (wt.%) in the composite cannot be directly determined. Instead, the extent of Ti incorporation is assessed using surface-sensitive techniques, such as XPS and STEM–EDX, which confirm the presence and distribution of TiO_2_ within the carbon matrix.

### 2.4. Morphological and Electrochemical Characterisation

#### 2.4.1. Morphological Characterisation

The morphology and composition of the samples were examined using scanning electron microscopy (SEM) coupled with energy-dispersive X-ray (EDX) analysis, using a Hitachi SU-8230 microscope equipped with an Oxford detector-analyser (Tokyo, Japan). Scanning Transmission Electron Microscopy (STEM) analysis was conducted using a Hitachi HD-2700 (Hitachi, Tokyo, Japan) microscope equipped with an Energy-Dispersive X-ray (EDX) detector (Oxford Instruments X-max 100 TLE, High Wycombe, UK), operating at 200 kV. Powder samples were dispersed in ethanol using a probe-type ultrasonic homogeniser and then deposited onto standard Cu TEM grids with Formvar and lacey carbon polymer films. Structural studies were performed, providing secondary electron images (surface electron detector, SEM), Z-contrast images (HAADF—High Angle Annular Dark Field, ZC-mode), and transmission images (Bright Field, TEM-mode). A fast Fourier transform (FFT) was applied to determine interplanar distances, followed by an inverse fast Fourier transform (IFFT), and interplanar distances at the atomic scale were calculated from the IFFT profiles. The Raman studies were carried out at room temperature using a LabRam HR800 (Horiba Jobin-Yvon, Palaiseau, France) confocal micro-Raman spectrometer. All the Raman spectra were generated by exposing the specimens for 600 s to a 0.27, 0.003/0.8 mW, 532 nm wavelength green excitation laser and dispersing the sample-emitted signal onto the CCD detector using a 600 lines/mm grating. A nitrogen adsorption analyser was used to assess surface area and pore parameters (TriStar Plus, Micromeritics, Norcross, GA, USA). X-ray diffraction (XRD) measurements for all samples were performed over 5–90° on a Rigaku SmartLab X-Ray Diffractometer (Rigaku Corporation, Tokyo, Japan) using Cu Kα radiation (λ = 0.154060 nm) at room temperature. Phase identification was performed using the International Centre for Diffraction Data (ICDD) PDF-2 database. Chemical composition was analysed using Kratos Axis Ultra HSA X-ray Photoelectron Spectroscopy (XPS) (Kratos Analytical Ltd., Manchester, UK) with a 15 kV X-ray source. UV–Vis diffuse reflectance spectra were recorded for amorphous TiO_2_ and the TiO_2_-containing composites using a JASCO V-770 spectrophotometer (Tokyo, Japan) equipped with an integrating sphere, and the optical band-gap energies were estimated according to the Kubelka–Munk theory.

#### 2.4.2. Electrochemical Characterisation (3-Electrode Setup)

- Glassy Carbon (GC) electrode preparation

The immobilisation of the carbon material onto the electrode was accomplished using the method previously described by Brandão et al. [23,24]. In summary, 5 mg of carbon was dispersed in 950 µL of N,N-Dimethylformamide (DMF, 99.8%, Sigma Aldrich), along with 10 µL of Nafion^®^ 117 (~5%, Sigma Aldrich, MO, USA). The mixture was ultrasonicated for 2 h to achieve a homogeneous dispersion of the material. The amount of carbon coated on the GC electrode was determined by averaging several measurements, ensuring uniformity in the material and Nafion dispersion in DMF. The suspension was then carefully applied to the GC electrode surface using a micropipette and allowed to dry at room temperature. Once dried, the electrode was assembled into the electrochemical cell and immersed in the eutectic mixture.

The choice of the ethaline-based DES as electrolyte is central to the objectives of this work. Unlike aqueous electrolytes, DES systems exhibit higher viscosity, asymmetric ion size, and strong hydrogen-bonding networks, which significantly influence ion transport and interfacial charge-storage processes. These characteristics impose diffusion limitations, making the electrochemical response more sensitive to pore structure, surface functionality, and electrode architecture.

In addition, the non-aqueous nature and wider electrochemical stability window of the DES minimise contributions from parasitic reactions, allowing a clearer assessment of capacitive and pseudocapacitive processes associated with the TiO_2_–carbon composites. Importantly, this electrolyte environment also affects the system’s light-assisted behaviour. Under these conditions, charge storage is dominated by surface-confined processes rather than bulk redox reactions, and photogenerated charge carriers in TiO_2_ can be more effectively stabilised at the TiO_2_–carbon interface. This contributes to the enhanced electrochemical response observed under illumination, including increased anodic currents and extended discharge times.

The mass loading of the active material on the glassy carbon electrode was kept constant for all measurements and was approximately 0.35–0.40 mg cm^−2^, as determined gravimetrically from multiple independent depositions. All electrochemical data reported herein are normalised to the active material mass. The silver wire used as a quasi-reference electrode provides a stable potential under the experimental conditions employed; however, all potentials discussed are referenced to the ferrocene/ferrocenium (Fc/Fc^+^) redox couple, measured in the same electrolyte and cell configuration. This internal reference allows reliable comparison of electrochemical features within the studied potential window.

- Electrochemical characterisation of a half-cell setup

The electrochemical measurements were conducted using a three-electrode cell connected to a computer-controlled VSP-300 multichannel potentiostat from BioLogic, operated through EC-Lab V11.26 software. The three-electrode setup included a GC electrode as the working electrode, a graphite counter electrode, and a silver wire pseudo-reference electrode. Before each measurement, the working electrode was polished according to methods previously described by several authors [23,24,25].

The electrochemical experiments were carried out at 30 °C, with temperature regulation provided by a thermostatic bath. Voltammetric experiments were performed at a scan rate of 50 mV s^−1^, starting at 0 V and sweeping to 1 V.

Electrochemical impedance spectroscopy (EIS) was conducted in a three-electrode electrochemical cell using a 10 mV AC perturbation over a frequency range of 20 kHz to 0.1 Hz, at a fixed potential of 0.5 V vs. Ag. Prior to each measurement, the working electrode was held at 0.5 V vs. Ag for at least 10 min to ensure a steady electrochemical state. This potential was selected as a midpoint within the active charge-storage window to enable comparison across electrode formulations.

Galvanostatic charge/discharge curves were recorded at a current density of 1 A g^−1^. The specific capacitance in the three-electrode configuration was calculated from the galvanostatic discharge curves, following the method outlined by Stoller et al., using Equation (1) [26]:

(1) $$C=\frac{I∆t}{m∆V}$$

where *I* is the discharge current (A), Δ*t* is the discharge time (s), Δ*V* is the potential window (V), and *m* is the weight of the carbon material in the electrode.

Because the present electrochemical study was performed in a three-electrode configuration to evaluate the intrinsic behaviour of the working electrode, the principal quantitative metric reported is the specific capacitance. Energy density and power density were not calculated because these parameters are more appropriately derived from two-electrode full-cell measurements that represent practical device operation.

To evaluate the effect of light on the electrochemical response, measurements were performed under controlled illumination and in the dark. Illumination was provided by a 60 W LED light source, positioned 10 cm from the electrochemical cell within an enclosed setup to ensure consistent exposure (Figure S2, Supporting Information). The light source was operated under constant conditions throughout all experiments. A corresponding dark configuration was achieved by fully shielding the cell from ambient light. The LED source provides broad-spectrum visible irradiation; therefore, the observed photoresponse reflects the overall light-induced behaviour of the TiO_2_-containing electrodes under these conditions.

To accurately define the potential window employed for electrochemical characterisation and ensure meaningful interpretation of capacitive and pseudocapacitive behaviour, we measured cyclic voltammograms using the ferrocene/ferrocenium (Fc/Fc^+^) redox couple in ethaline under identical conditions (electrolyte, temperature, electrode configuration) to those used in the TiO_2_-C composite studies. The Fc/Fc^+^ redox pair exhibited a well-defined, reversible response, with peak potentials centred at 0.54 V (reduction) and 0.63 V (oxidation) versus the Ag quasi-reference electrode. The consistent peak current ratios, narrow peak separations, and linear Randles–Ševčík behaviour confirmed the electrochemical reversibility and diffusion-controlled nature of the redox process. This measurement validates the stability of the 0–1 V window employed throughout the study and provides a reference scale to contextualise the observed surface redox contributions from the TiO_2_-based electrodes. Full characterisation data and analysis are provided in the Supporting Information (Figures S3 and S4).

## 3. Results

The morphological characterisation and the elemental composition of the biocarbons were studied to determine how their capacitive behaviour was affected, considering the distinct fish waste precursors. The electrochemical investigation included CV, EIS, and GCD measurements to validate the electrode’s capacitive performance. The characteristics of the biocarbon composed of prawn chitin (PC) and blue shark gelatine (BSG) will be compared with the composites with TiO_2_: PC+TiO_2_, and BSG+TiO_2_.

### 3.1. Nitrogen Adsorption/Desorption Isotherms

**Figure 1:**
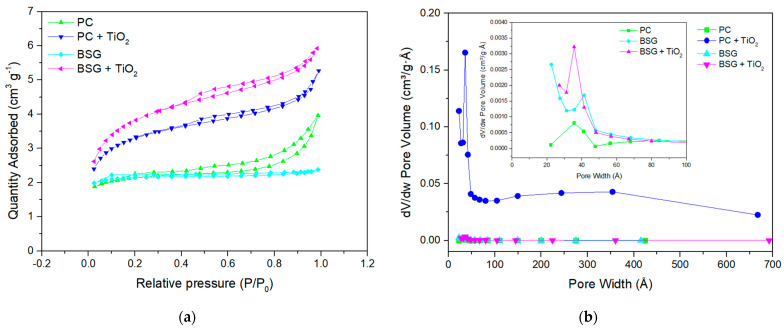
Relationship between the adsorbed and desorbed volumes of N_2_ by the fish waste-based carbons (carbonised at 1000 °C for 1 h) with and without TiO_2_ attachment and the relative pressure (**a**) and the pore size distribution (**b**).

Adsorption–desorption isotherms are used to obtain qualitative and quantitative information about a material’s surface area and pore structure. The adsorption–desorption isotherms of the fish waste-based carbons (with and without TiO_2_) were obtained and are presented in Figure 1a. The isotherms exhibit different characteristics; however, they show similar trends and are classified as Type IV according to the Brunauer–Emmett–Teller (BET) classification [27]. The main characteristic feature is their hysteresis loop, which may correlate to the capillary condensation process in the mesopores. Figure 1b presents the pore size distribution of the studied samples, showing a sharp peak at ~35 Ả for PC, ~50 Ả for PC+TiO_2_, ~40 Ả for BSG and ~36 Ả for BSG+TiO_2_.

The textural properties of the materials play a critical role in determining their electrochemical performance, particularly in systems where electrolyte properties limit ion transport. The increase in specific surface area observed after TiO_2_ incorporation is expected to enhance the number of electrochemically active sites available for charge storage. In addition, the pore-size distribution is particularly important. While micropores contribute to high surface area, they may not be fully accessible to electrolyte ions, especially in deep eutectic solvents, which typically exhibit higher viscosity and larger effective ion sizes than aqueous electrolytes.

In this context, the presence of mesopores in the TiO_2_-containing composites is beneficial, as they facilitate electrolyte penetration and improve ion transport within the electrode structure. The combination of micropores (for charge storage) and mesopores (for ion transport pathways) results in hierarchical porosity that favours capacitive behaviour. This structural configuration is consistent with the improved electrochemical performance observed for the TiO_2_-modified materials, indicating that the interplay between surface area and pore architecture is a key factor governing charge storage in these systems.

Table 1 summarises the BET analysis of the studied samples, in which the PC and BSG were already given by Brandão et al. [10,11]. The attachment of TiO_2_ to the surface against the carbons synthesised from the 3 distinct biomass sources showed a substantial increase in the specific surface area and pore diameter (Dp) of all the studied precursors, with a more expressive increase on the PC and BSG, which presented a small surface area before the TiO_2_ attachment.

The substantial increase in S_BET_ observed after TiO_2_ incorporation cannot be attributed solely to the intrinsic surface area of TiO_2_ but instead reflects a structural reorganisation of the carbon framework during in situ oxide formation. The anodic dissolution–hydrolysis process occurring in the DES medium promotes the deposition of TiO_2_ nanoparticles at defect sites, pore entrances, and interlayer regions of the carbon matrix. This spatially distributed oxide phase acts as a physical spacer, limiting the restacking of carbon layers while simultaneously inducing local framework erosion and pore widening.

Therefore, the original micropore-dominated carbons evolve toward a mesopore-enriched architecture, as evidenced by the marked shift in pore diameter and the pronounced increase in mesoporous volume. This hierarchical porosity is particularly favourable for ion-accessible surface area rather than purely geometric surface expansion, which explains the disproportionate increase in S_BET_ relative to the modest TiO_2_ loading.

### 3.2. SEM, STEM and EDX Analyses

**Figure 2:**
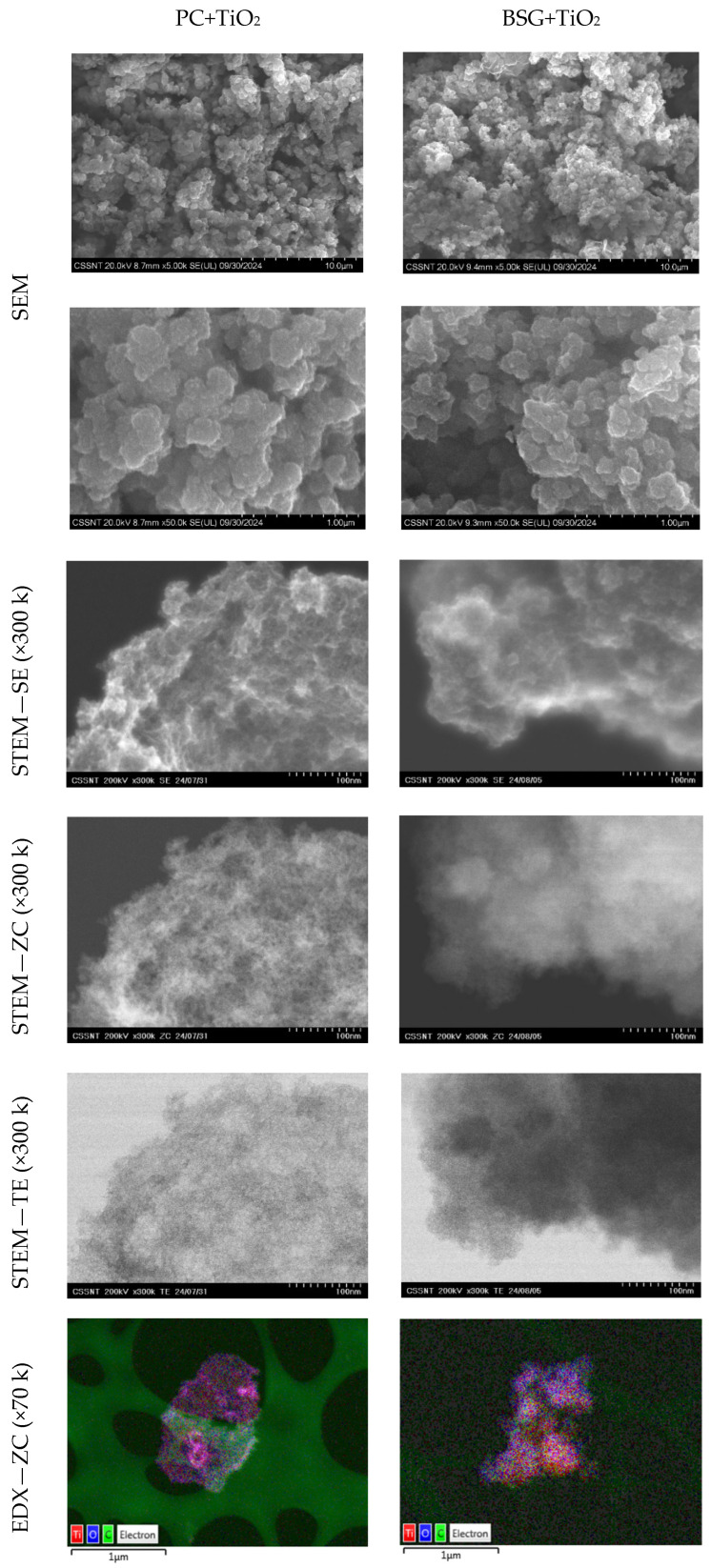
SEM, STEM (SE mode (×300)), ZC image (×300), TE mode (×300) and EDX mapping analysis (×70 k) for PC+TiO_2_ and BSG+TiO_2_.

The morphology of the fish waste-based carbons was assessed by SEM, UHR-STEM, and EDX analysis, and the results were compared with those of the TiO_2_ composites obtained using different precursors. Figure S5 presents the SEM analysis of the already published PC and BSG carbon-based materials [10,11,28]. EDX was also performed, showing carbon as the most abundant element, with traces of oxygen, nitrogen, phosphorus, calcium, and sulfur, as expected given the samples’ organic source. Figure 2 presents the SEM analysis of the three studied composites: PC+TiO_2_ and BSG+TiO_2_. The pore structure of the samples consists of several pores that expose a large, activated surface area. This shortens the ions’ diffusion paths, potentially improving the electrochemical properties of these materials [29]. The surface morphology and pore structure of the composite samples were confirmed by ultra-high-resolution scanning transmission electron microscopy (UHR-STEM). The images were obtained using different detectors: secondary electrons (STEM-SE), phase contrast (STEM-ZC), transmission electrons (STEM-TE), and EDX (EDX–ZC).

The presence of TiO_2_ is well defined by EDX analysis, as is the relevant pore structure, which can be quite beneficial for electrolyte ions to move to the carbon electrode (in the supercapacitor setup), thereby achieving a high specific capacitance and excellent performance [30]. Using this technique, the interplanar distance of 0.35 nm was determined for all composite samples, suggesting that they may share a similar crystallographic structure.

The structural features of prawn chitin and blue shark gelatin-derived carbons without the TiO_2_ attachment were already studied using Fourier Transform Infrared (FTIR). FTIR analysis of these carbon materials, carbonised at 1000 °C for 1 h, revealed characteristic absorption bands that confirm the presence of functional groups associated with oxygen and nitrogen, indicative of their biogenic origin. PC displayed bands corresponding to N-H bending at 1550 cm^−1^ and C-N stretching at 1380 cm^−1^, reflecting the nitrogen-doping effect inherent to chitin’s molecular structure [10]. For BSG, notable bands around 3300 cm^−1^ (N-H stretching) and 1600 cm^−1^ (C=O stretching) were observed, pointing to the presence of amide groups from the proteinaceous origin of gelatin [11].

The STEM images (Figure 2) provide direct evidence of the dispersion of TiO_2_ nanoparticles within the carbon matrix, with clear contrast between the inorganic phase and the carbon framework. Importantly, the corresponding EDX elemental mapping confirms the presence and spatial distribution of Ti, demonstrating that the TiO_2_ phase is uniformly distributed rather than segregated. This combined STEM–EDX analysis indicates that TiO_2_ nanoparticles are anchored onto and embedded within the conductive carbon network, forming an intimate TiO_2_–carbon interface. Such structural integration is essential for facilitating efficient charge transfer between the semiconductor and conductive phases.

### 3.3. Raman Analysis

**Figure 3:**
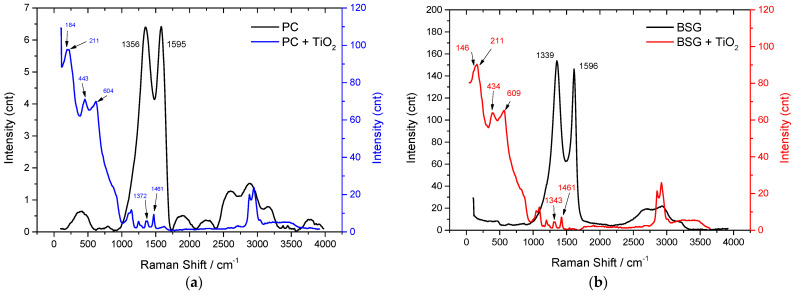
Raman analysis (full spectra) for PC and PC+TiO_2_ (**a**), BSG and BSG+TiO_2_ (**b**).

Raman spectroscopy was performed and presented in Figure 3 for PC, PC+TiO_2_, BSG, and BSG+TiO_2_. Raman spectroscopy was used not only to identify the vibrational features of TiO_2_, but also to assess the structural order/disorder of the carbon framework and the effect of oxide incorporation on the carbon matrix. The Raman analysis provides insight into the chemical structure and the interaction between TiO_2_ and the carbon matrix. For BSG, peaks were observed around 1240–1270 cm^−1^ (Amide III), 1450–1470 cm^−1^ (CH_2_/CH_3_ bending), and 1660–1690 cm^−1^ (Amide I), all of which are typical for proteins such as collagen. The 2600–3000 cm^−1^ region featured strong aliphatic C–H stretching vibrations, indicative of hydrocarbon chains from amino acid side groups. The persistence of these bands indicates that BSG retains heteroatom-containing organic functionalities, which may act as potential anchoring sites for TiO_2_ nanoparticles.

After TiO_2_ integration, the BSG+TiO_2_ composite displayed additional peaks corresponding to anatase TiO_2_, including modes at 146–211 cm^−1^ (Eg), 434 cm^−1^ (B1g and A1g + B1g), and 609 cm^−1^ (Eg), confirming the presence of the anatase crystalline phase. These bands are consistent with the anatase TiO_2_ phase and indicate successful incorporation of the oxide into the carbonaceous matrix. Organic bands such as Amide III (~1086–1273 cm^−1^), CH_2_/CH_3_ bending (~1343–1461 cm^−1^), Amide I (~1660 cm^−1^), and C–H stretches (~2863–3037 cm^−1^) remained evident, indicating that TiO_2_ attachment occurred without disrupting the primary carbon matrix. At the same time, slight changes in band shape and relative intensity suggest interfacial interactions between TiO_2_ and the organic/carbonaceous framework rather than simple physical mixing.

For PC, the Raman spectrum revealed bands at ~1356 cm^−1^ (C–H bending from CH_3_ and CH_2_ groups) and ~1595 cm^−1^ (C=O stretching in Amide I), corresponding to the N-acetylglucosamine units of the chitin polymer. In addition, these two bands can be assigned to the characteristic D and G bands of carbon, where the D band is related to defect-induced vibrations and the G band corresponds to the in-plane stretching of sp^2^-hybridised carbon domains. The high-frequency region between ~2604–3171 cm^−1^ confirmed the presence of aliphatic C–H stretches.

In the PC+TiO_2_ composite, characteristic anatase bands appeared between 184 and 211 cm^−1^ (Eg), 443–542 cm^−1^ (B1g and A1g + B1g), and at 604 cm^−1^ (Eg). Organic bands were also preserved: Amide III (~1087–1272 cm^−1^), CH_2_/CH_3_ bending (~1338–1461 cm^−1^), Amide I (~1618 cm^−1^), and aliphatic C–H stretches (~2869–3039 cm^−1^). These findings demonstrate a successful TiO_2_ integration into the carbon network without significant degradation of the original chitin structure. Compared with pristine PC, the carbon-related bands in PC+TiO_2_ are broader, less resolved, and slightly shifted, indicating that TiO_2_ incorporation perturbs the local carbon structure and modifies the degree of carbon framework ordering.

The spectra showed distinct D and G bands, located approximately at 1356 cm^−1^ and 1595 cm^−1^ (for PC) and 1339 cm^−1^ and 1596 cm^−1^ (for BSG). The intensity ratio of these bands (I_D_/I_G_) indicates the degree of disorder and graphitic domains within the carbon structure. After baseline correction, the I_D_/I_G_ ratio provides a semi-quantitative measure of defect density and graphitic ordering in the carbon materials. PC and BSG exhibit high I_D_/I_G_ ratios of 1.03 and 1.13, respectively, indicating a high structural disorder typical of nitrogen-doped carbons. The slightly higher I_D_/I_G_ value of BSG suggests a higher density of structural defects and a lower degree of graphitic ordering compared with PC. Such a defect-rich, partially graphitised structure is beneficial for electrochemical applications, as defect sites and surface functionalities can increase the number of active sites and improve electrolyte accessibility. At the same time, graphitic domains contribute to electronic conductivity. These findings corroborate the morphological and electrochemical data, further demonstrating the suitability of these materials for high-performance supercapacitor applications.

Moreover, the Raman spectra, indicating a balance between amorphous and graphitic carbon phases, suggest a surface structure that supports uniform TiO_2_ distribution. The moderate level of disorder, as reflected in the ID/IG ratio, may provide a heterogeneous surface that promotes better adhesion of TiO_2_ nanoparticles, thereby improving the composite’s capacitance and charge/discharge performance. Therefore, the Raman results are consistent with strong interfacial coupling between TiO_2_ and the carbon substrates, which may contribute to the improved electrochemical behaviour observed for the composite electrodes. Overall, the Raman analysis confirms preservation of the carbon framework after TiO_2_ incorporation, while also revealing modifications in structural disorder, graphitic character, and interfacial interactions within the resulting composites.

### 3.4. XPS Analysis

**Figure 4:**
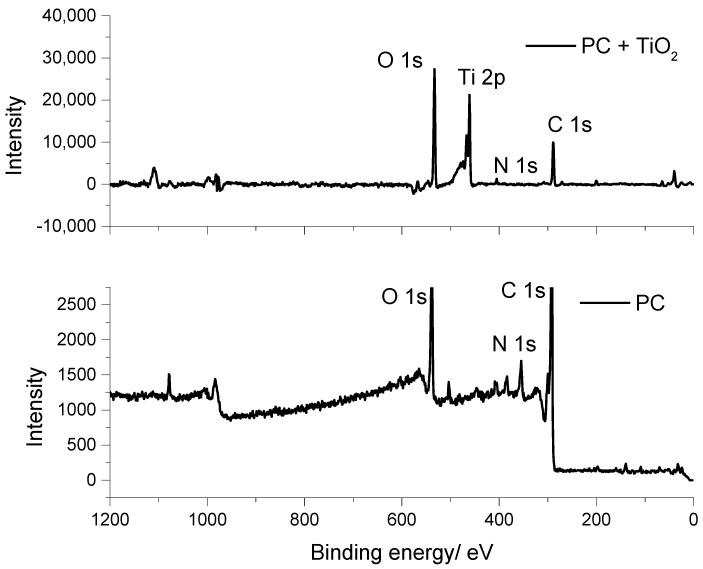
XPS survey overall spectra for PC+TiO_2_ (**upper**) and PC (**lower**).

**Figure 5:**
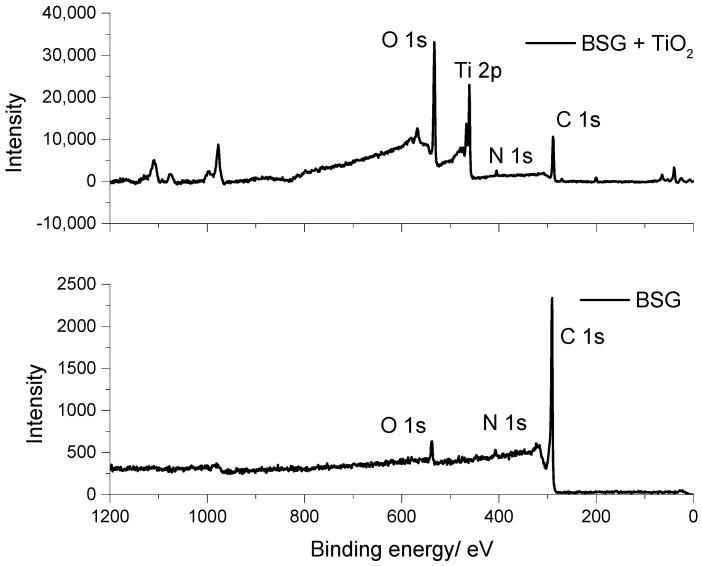
XPS survey overall spectra for BSG+TiO_2_ (**upper**) and BSG (**lower**).

X-ray photoelectron spectroscopy (XPS) was used to examine the surface composition of the carbon materials before and after TiO_2_ incorporation. The survey spectra of PC, PC+TiO_2_, BSG, and BSG+TiO_2_ are shown in Figure 4 and Figure 5, while the corresponding surface atomic percentages are summarised in Table 2. For the pristine carbon materials, the main contributions arise from C 1s, N 1s, and O 1s, consistent with the biomass-derived nature of the carbons and the presence of residual heteroatom-containing functionalities. In contrast, the TiO_2_-containing composites additionally show a clear Ti 2p contribution, confirming the successful attachment of the oxide phase to the carbon matrix.

The atomic-composition data reveal significant changes following TiO_2_ incorporation. In both precursor systems, the C 1s contribution decreases markedly, from 78.4 at.% to 50.4 at.% for PC and from 81.4 at.% to 41.2 at.% for BSG, while the O 1s contribution increases from 14.3 at.% to 35.1 at.% for PC and from 4.1 at.% to 34.6 at.% for BSG. At the same time, Ti 2p becomes clearly detectable, reaching 12.6 at.% in PC+TiO_2_ and 21.1 at.% in BSG+TiO_2_. These compositional changes are fully consistent with the successful formation of TiO_2_ on the carbon surface and indicate that the oxide phase substantially modifies the surface chemistry of the original carbons. The higher Ti 2p contribution observed for BSG+TiO_2_ suggests a greater surface abundance of Ti-containing species in this composite.

A more detailed interpretation of the XPS results provides insight into the chemical environments present at the composite surface. The carbon signal is predominantly associated with graphitic carbon (C–C/C=C), along with oxygen-containing functionalities such as C–O and C=O, which are typical of biomass-derived carbons and can act as anchoring sites for oxide nanoparticles. The strong increase in the O 1s signal after TiO_2_ incorporation is consistent with the introduction of oxygen-rich TiO_2_ species, while also reflecting contributions from surface hydroxyl and carbon–oxygen groups. In parallel, the Ti 2p signal is characteristic of Ti^4+^, confirming that titanium is present in the oxidised state expected for TiO_2_.

Taken together, these results support a strong interfacial interaction between TiO_2_ and the carbon matrix. In particular, the coexistence of Ti–O environments with carbon-bound oxygen functionalities suggests that the interface is mediated by oxygen-containing surface groups, which likely facilitate the anchoring and stabilisation of TiO_2_ nanoparticles on the carbon framework. Although the present survey spectra do not allow an unambiguous assignment of direct Ti–O–C bonding, the XPS data are consistent with oxygen-mediated interfacial coupling between TiO_2_ and the carbon surface. Such coupling is expected to favour charge transfer across the TiO_2_–carbon interface, in agreement with the improved electrochemical response observed for the composite electrodes.

A more detailed analysis of the XPS spectra provides insight into the surface chemical states and possible interfacial interactions between TiO_2_ and the carbon matrix. The C 1s spectra indicate the presence of graphitic carbon (C–C/C=C) and oxygen-containing functional groups, such as C–O and C=O, which are typical of biomass-derived carbons and are known to act as anchoring sites for metal oxides. The O 1s spectra show a significant increase in oxygen contribution after TiO_2_ incorporation, which can be attributed to lattice oxygen from TiO_2_ as well as surface hydroxyl and carbon–oxygen species [31,32,33].

The Ti 2p region confirms the presence of Ti^4+^, characteristic of TiO_2_, indicating that the oxide phase has formed and is stable on the carbon surface. The coexistence of Ti–O and C–O environments suggests that the interaction between TiO_2_ and the carbon matrix is likely mediated through oxygen-containing functional groups [31]. Although the formation of direct Ti–O–C bonds cannot be unambiguously confirmed without high-resolution spectral deconvolution or complementary techniques, the XPS results are consistent with strong interfacial coupling between TiO_2_ and the carbon framework.

### 3.5. UV–Vis Diffuse Reflectance and Band-Gap Analysis

**Figure 6:**
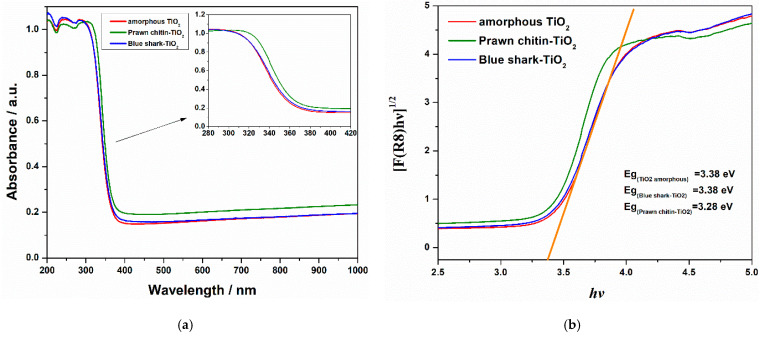
(**a**) UV–Vis diffuse reflectance spectra of amorphous TiO_2_, prawn chitin-TiO_2_ and blue shark-TiO_2_; (**b**) plot of [F(R∞)hν]^1/2^ vs. hν for band gap calculation.

To further support the discussion of the light-responsive behaviour of the TiO_2_-containing materials, UV–Vis diffuse reflectance spectroscopy (DRS) was performed for amorphous TiO_2_, PC+TiO_2_, and BSG+TiO_2_ (Figure 6).

The band gap energy (Eg) of TiO_2_ and for the prawn chitin-TiO_2_ and blue-shark-TiO_2_ were extracted from the DRS spectra by extrapolating the linear region of the plot [F(R∞)hν]^1/2^ vs. hν according to the Kubelka-Munk theory in the assumption of the interband direct transition where F(R∞) is the Kubelka-Munk function, R∞ is the absolute reflectance of sample, hν is the photon energy and Eg the band gap energy. The band gap for amorphous TiO_2_ was found to be 3.38 eV, which aligns well with the values reported in the literature [34,35]. As expected, this value exceeds the band gaps of crystalline anatase (~3.2 eV) and rutile (~3.0 eV), further confirming that structural disorder shifts the absorption edge to higher energies than those of the more ordered crystalline phases [34,36].

### 3.6. XRD Analysis

The XRD patterns of PC+TiO_2_ and BSG+TiO_2_ (Figure S6, previously demonstrated by Brandão et al. [10,11,28]) reveal a predominantly amorphous carbon background, consistent with biomass-derived carbons carbonised at high temperature. Superimposed on this broad halo, weak but well-defined diffraction peaks associated with TiO_2_ are observed.

Although the TiO_2_ was not subjected to a post-synthesis calcination step, the appearance of discrete reflections indicates the presence of short-range crystalline domains with limited coherence length rather than fully developed bulk crystallinity. This interpretation is consistent with nanocrystalline anatase domains embedded within an amorphous or poorly ordered matrix, as further supported by Raman spectroscopy.

Importantly, the absence of sharp graphite-related reflections confirms that the carbon matrix remains largely amorphous, while the TiO_2_ phase contributes localised crystallinity. Such a mixed amorphous–nanocrystalline structure is advantageous for capacitive applications, as it maximises surface defect density and interfacial charge-storage sites without imposing the long-range electron-transport limitations typically associated with bulk oxide crystallinity.

### 3.7. Electrochemical Analysis

#### 3.7.1. CV and GCD Analysis

**Figure 7:**
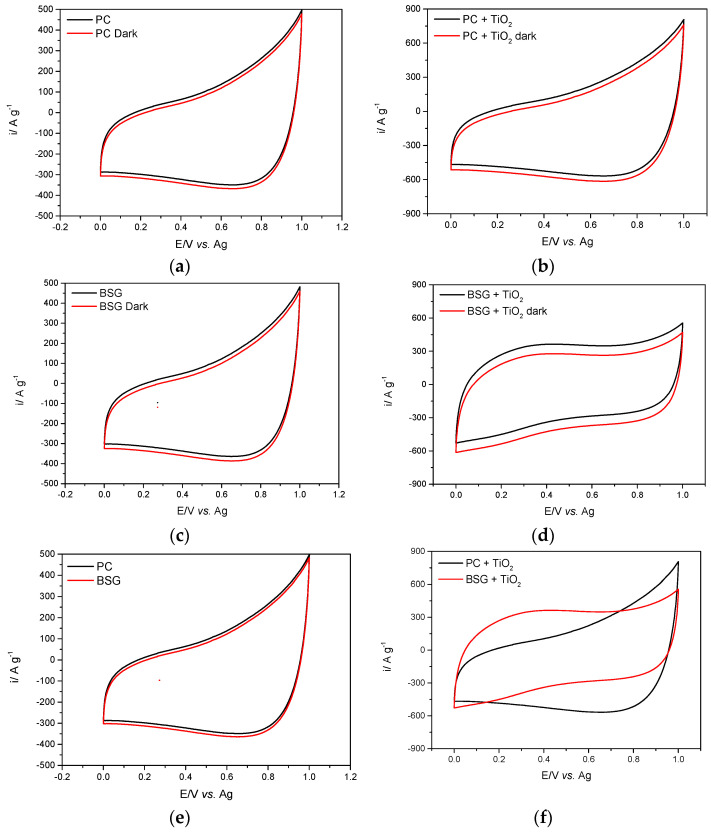
Cyclic voltammetric curves (30 °C, ethaline, 50 mV s^−1^) for PC and PC+TiO_2_ (**a**,**b**), BSG and BSG+TiO_2_ (**c**,**d**), and the comparison between PC and BSG (**e**) and the two composite materials in (**f**).

CV analysis was performed to study the electrochemical performance of the carbons and composites within the accessible potential window vs. a silver wire reference electrode. The cyclic behaviour of the carbon/composite samples is presented in Figure 7 for PC and PC+TiO_2_ (Figure 7a,b), BSG and BSG+TiO_2_ (Figure 7c,d), and the comparison between the two composite materials in Figure 7e,f. The electrochemical analysis in the dark is also presented in Figure 7a–d.

The carbon material electrodes exhibited pseudo-rectangular voltammetric profiles, showing typical electric double-layer capacitive behaviour [37] increased, indicating that the presence of TiO_2_ enhances the electrochemical response in the system. Even though the three composite samples present a similar cathodic current, PC+TiO_2_ presents the highest anodic current, followed by BSG+TiO_2_, which strictly correlate with the specific surface area and pore size diameter.

The carbon material electrodes exhibited pseudo-rectangular voltammetric profiles, showing typical electric double-layer capacitive behaviour [37]. All composite samples exhibit increased current responses, indicating that TiO_2_ enhances the electrochemical response in the system. TiO_2_ exhibits pseudocapacitive behaviour, in which redox reactions occur. In contrast to pure EDLCs, pseudocapacitive materials store energy via surface- or near-surface redox reactions, thereby increasing charge storage. TiO_2_, particularly in its nanostructured form, often exhibits pseudocapacitance, which complements the carbon material’s EDLC.

Even though the three composite samples present a similar cathodic current, PC+TiO_2_ presents the highest anodic current, followed by BSG+TiO_2_, which strictly correlate with the specific surface area and pore size diameter. The specific surface area of a material directly correlates with its capacity to store charge in the electric double layer. A higher SSA allows more ions from the electrolyte to accumulate at the porous electrode surface, thereby increasing the current response. The trend suggests that PC+TiO_2_ has the highest SSA, indicating more active charge-accumulation sites, while coupled with mesoporous biocarbon with pores ranging from 2 to 50 nm is especially critical because it may balance, providing surface area and allowing rapid electrolyte ion diffusion. PC+TiO_2_ might have a more favourable pore structure, facilitating faster ion transport and thereby increasing the anodic current. The interaction between TiO_2_ and the biocarbon matrix can also affect electrochemical performance; consequently, PC+TiO_2_ may exhibit a synergistic interaction, enabling efficient charge transfer between TiO_2_ and the carbon material.

BSG+TiO_2_ also exhibits good performance, but to a lesser extent, indicating that the choice of carbon precursor (PC or BSG) for TiO_2_ nanoparticle decoration and its interaction with the electrolyte are critical for optimising the material’s electrochemical performance. Cyclic voltammetry (CV) results highlight an apparent enhancement in electrochemical performance with the inclusion of TiO_2_ across all composite samples, while also revealing the impact of dark conditions on their behaviour. Under illumination, the cyclic voltammetry profiles exhibit a notable increase in the anodic current. In contrast, the cathodic current response remains comparable or slightly reduced relative to dark conditions, suggesting asymmetric photoelectrochemical behaviour potentially linked to directional charge transport or surface reaction kinetics, underscoring the light-sensitive properties of the TiO_2_-based composites. This decrease suggests that the photogenerated charge carriers in TiO_2_ play a significant role in amplifying the electrochemical response under illumination, contributing to both the electric double-layer capacitance (EDLC) of the carbon matrix and the pseudocapacitive behaviour of TiO_2_. The reduction in current in the dark is consistent across all samples, with PC+TiO_2_ still maintaining the highest anodic current, followed by BSG+TiO_2_. This behaviour underscores the critical role of TiO_2_’s light-responsive properties, which synergistically interact with the biocarbon matrix’s high surface area and favourable pore structure to enhance charge storage capabilities. Even in the absence of light, the materials retain their inherent electrochemical functionality, albeit at a reduced level, suggesting that the TiO_2_-carbon interaction facilitates stable charge storage. These findings emphasise the dual-mode potential of these composites, performing reliably in dark conditions while achieving superior performance under light, making them promising candidates for versatile energy storage systems. Minor variations under illumination were also observed for the carbon-only electrodes (PC, BSG), potentially due to photothermal effects or localised charge redistribution at functionalised carbon sites. However, these effects were much less pronounced than in TiO_2_-containing composites, confirming that the TiO_2_ component primarily governs the light-responsive behaviour.

To clarify the electrochemical origin of the increased current response observed in the composites, it is important to distinguish between classical bulk redox and surface-confined pseudocapacitive processes. While Ti^4+^/Ti^3+^ bulk redox typically occurs at more negative potentials in aqueous systems, in deep eutectic solvents such as ethaline, it is unlikely to occur within our applied potential window (0–1 V vs. Ag). Instead, the pseudocapacitive enhancement is attributed to surface-mediated processes, including shallow electron trapping, redox activity at oxygen vacancies, reversible transformations of surface hydroxyls, and Ti^3+^-related surface states. These mechanisms are supported by recent studies on structurally activated TiO_2_ nanomaterials, which demonstrate pseudocapacitive behaviour in non-aqueous and hybrid systems even without full Ti^4+^/Ti^3+^ conversion [38]. In the studied composites, the conductive carbon matrix likely promotes charge separation and storage by stabilising photogenerated carriers or surface charges, thereby enhancing the CV current response in a manner consistent with pseudocapacitive behaviour.

The CV profiles of the TiO_2_-containing composites do not display distinct redox peaks, indicating that classical bulk Ti^4+^/Ti^3+^ insertion-type reactions are not dominant under the present conditions. Instead, the electrochemical response is characteristic of a combination of electric double-layer capacitance from the carbon matrix and surface-confined pseudocapacitive contributions associated with the TiO_2_ phase.

These pseudocapacitive processes are attributed to fast surface redox reactions and interfacial charge transfer at the TiO_2_–carbon interface, rather than diffusion-limited bulk transformations. The absence of sharp redox features and the quasi-rectangular CV shape support this interpretation. Under illumination, the contribution of TiO_2_ becomes more pronounced, as photoinduced charge carriers generated within the semiconductor are partially separated and stabilised at the interface with the conductive carbon matrix, leading to an increase in current response.

**Figure 8:**
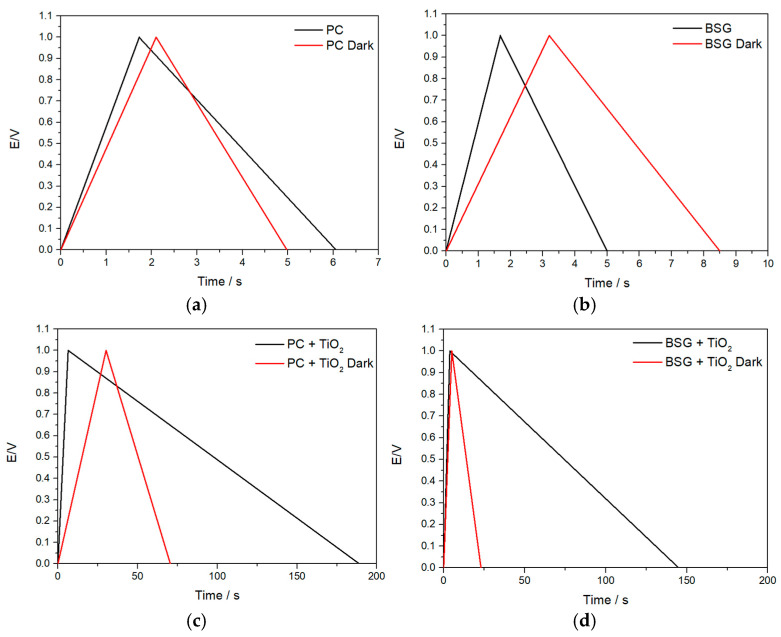
GCD curves in ethaline at 30 °C (1st cycle) of PC (light and dark) (**a**), BSG (light and dark) (**b**), PC+TiO_2_ (light and dark) (**c**), and BSG+TiO_2_ (light and dark) (**d**).

The GCD curves for the carbons/composites samples in the presence and absence of natural light are presented in Figure 8a–d. The GCD profiles of the pristine carbon electrodes (Figure 8a,b) display a quasi-triangular shape, consistent with predominantly electric double-layer capacitive behaviour. In contrast, the TiO_2_-containing composites (Figure 8c,d) exhibit non-ideal charge–discharge profiles, particularly under illumination, with a nearly linear charging branch and an angled discharge branch. This behaviour is attributed to the coexistence of electric double-layer charging and light-assisted surface/interfacial pseudocapacitive processes at the TiO_2_–carbon interface, rather than to purely ideal capacitive storage. In addition, the viscous, highly structured DES electrolyte contributes to interfacial polarisation and slower ion transport, further accentuating evolutions from the symmetric triangular shape typically observed in conventional aqueous EDLC systems [39].

Table 3 presents the specific capacitance (calculated using Equation (1), as shown in Figure 8) and the capacitance retention after 1000 and 10,000 cycles for different composite samples. The electrochemical performance is therefore discussed primarily in terms of specific capacitance and cycling stability, since these quantities directly reflect the behaviour of the active material in the three-electrode configuration employed here. In contrast, device-level metrics such as energy density and power density were not considered, as their calculation from half-cell data would not provide a rigorous basis for comparison with practical supercapacitor devices. The comparison with aqueous and organic electrolytes is also presented.

To better contextualise the electrochemical performance of the present fish-waste-derived TiO_2_@C composites, comparison with both bare TiO_2_ and representative carbon–TiO_2_ systems is necessary. Bare TiO_2_ is an attractive electrode material due to its chemical stability, low cost, and photoactivity; however, its supercapacitor performance is often constrained by poor electronic conductivity and relatively low specific capacitance [40]. For this reason, carbon incorporation is commonly used to improve electrical transport, increase the electrochemically accessible surface area, and facilitate interfacial charge storage. This trend is consistent with the present results, where TiO_2_ incorporation increases the specific capacitance from 15 ± 2 to 54 ± 3 F g^−1^ for PC and from 7 ± 1 to 49 ± 5 F g^−1^ for BSG, while maintaining good long-term stability (91% and 85% retention after 10,000 cycles for PC+TiO_2_ and BSG+TiO_2_, respectively). Literature reports also show that conventional carbon–TiO_2_ composites can achieve higher capacitance values than those obtained here, particularly in aqueous electrolytes and in systems specifically optimised for high-rate charge storage [42]. Therefore, the main advantage of the present materials lies not in achieving record capacitance but in combining marine-waste-derived carbon precursors, in situ TiO_2_ deposition in a deep eutectic solvent, long-term cycling durability, and light-assisted capacitive behaviour under non-aqueous DES conditions.

PC+TiO_2_ increases capacitance, from 15 ± 2 F g^−1^ for pure PC to 54 ± 3 F g^−1^ for PC+TiO_2_. This increase (almost 3 times) may likely be due to the combined effects of TiO_2_’s pseudocapacitive contribution and the enhanced surface area. BSG+TiO_2_ shows a greater relative improvement, increasing from 7 ± 1 F g^−1^ to 49 ± 5 F g^−1^ (7 times higher). Even though BSG initially had the lowest surface area and capacitance, TiO_2_ transforms its electrochemical properties, making it comparable to PC+TiO_2_. The strong correlation between increased SBET and C across all samples highlights the importance of surface area and TiO_2_’s role in enhancing the charge-storage capability of carbon materials. The significant increase in capacitance for PC and BSG after TiO_2_ incorporation demonstrates that TiO_2_ enhances the overall electrochemical response, likely through pseudocapacitance and improved ion accessibility within the porous structure.

PC+TiO_2_ retains 97% at 1000 cycles and 91% at 10,000 cycles, showing slightly reduced but still excellent cycling stability. BSG+TiO_2_, despite having a lower initial capacitance, demonstrates 95% retention at 1000 cycles and a remarkable 85% at 10,000 cycles. This long-term cycling stability suggests that BSG+TiO_2_ could be a highly durable material for applications requiring extensive cycling. Pure PC retains 92% of its capacitance after 1000 cycles, demonstrating good stability but not as high as that of the PC+TiO_2_ composite. BSG have lower initial retention (71% at 1000 cycles), reflecting its less robust cycling stability without TiO_2_ modification.

The long-term cycling stability of the TiO_2_-containing composites was evaluated over 10,000 charge–discharge cycles, showing high capacitance retention and stable electrochemical response. This number of cycles is consistent with standard protocols for assessing the durability of supercapacitor electrode materials, particularly in three-electrode configurations, where the intrinsic stability of the active material is evaluated.

The observed stability can be attributed to several factors. First, the carbon matrix provides a mechanically robust and electrically conductive framework that maintains structural integrity during repeated cycling. Second, the TiO_2_ nanoparticles are strongly anchored to the carbon surface, as evidenced by structural and XPS analyses, thereby helping prevent detachment or aggregation during operation. Finally, the deep eutectic solvent electrolyte exhibits high electrochemical stability within the selected potential window, minimising degradation during extended cycling.

These combined effects contribute to the stable capacitive behaviour observed, indicating that the TiO_2_–carbon composites are suitable for long-term electrochemical operation under the studied conditions. A more quantitative analysis of the electrochemical response further highlights the effect of TiO_2_ incorporation and illumination on charge-storage behaviour. The cyclic voltammetry curves of the TiO_2_-containing composites exhibit increased current response and a more pronounced deviation from an ideal rectangular shape compared to the parent carbon materials, indicating the contribution of additional surface-controlled pseudocapacitive processes. This is consistent with the galvanostatic charge–discharge results, in which the TiO_2_-modified electrodes exhibit longer discharge times at the same current density, directly reflecting their higher specific capacitance.

In addition, the cycling stability data demonstrate that the composites retain a high fraction of their initial capacitance over extended cycling, confirming the structural stability of the TiO_2_–carbon interface under repeated charge–discharge conditions. The enhancement observed under illumination is also reflected quantitatively in the increased anodic currents and extended discharge profiles, indicating that light contributes to additional charge accumulation rather than merely affecting the system’s kinetics.

These results collectively indicate that the electrochemical behaviour of the TiO_2_-containing composites arises from a combination of electric double-layer capacitance from the carbon framework and surface-confined pseudocapacitive contributions from the TiO_2_ phase. The quantitative differences observed between the pristine carbons and the TiO_2_-modified materials therefore support the role of the semiconductor component in enhancing charge storage, particularly under illumination conditions.

PC+TiO_2_ emerges as the best-performing material, offering a high specific capacitance and excellent long-term cycling stability, making it highly suitable for energy storage devices like supercapacitors. The overall trend suggests that TiO_2_ is a key component in boosting the specific surface area and the charge storage capacity of carbon-based electrodes while enhancing their durability under repeated cycling.

The higher specific capacitance was obtained for PC+TiO_2_ at a current density of 1 A g^−1^. The higher specific capacitance value can be associated with the higher specific surface area of 412 ± 2 m^2^ g^−1^, which allows more Cl^−^ ions from the eutectic electrolyte to contact the pores. The higher specific capacitance probably originated from enhanced surface properties (elemental composition and functional groups).

The DES-based electrochemical response should be interpreted in light of the transport limitations inherent to DESs. Compared with aqueous electrolytes, DES media generally exhibit higher viscosity, lower ion mobility, and a more structured ionic environment, all of which reduce ion transport rates within porous electrodes and limit access to the full internal surface area. Among common choline chloride-based DESs, ethaline is considered one of the more favourable systems because of its comparatively lower viscosity and higher conductivity; however, it still imposes substantially greater transport constraints than aqueous electrolytes [43].

In the present materials, the electrochemical performance is therefore not limited by a single factor, but by the combined influence of electrolyte viscosity, effective ion size/ionic structuring, and electrode wetting. The hierarchical porosity generated after TiO_2_ incorporation is beneficial because mesopores facilitate electrolyte penetration and shorten diffusion pathways, whereas micropores may remain only partially accessible in the viscous DES medium. In addition, slower wetting of the porous electrode surface is expected to reduce the ion-accessible area and contribute to interfacial impedance. Consequently, the moderate capacitance values observed here are consistent with the use of a DES electrolyte. They should be understood as the result of constrained ion transport rather than solely as a lack of electrode activity [44].

The improved electrochemical performance of the TiO_2_-containing composites can also be correlated with their textural properties. The increase in specific surface area increases the number of electrochemically active sites for charge storage, while the pore-size distribution plays a critical role in ion transport. In particular, mesopores facilitate electrolyte penetration and reduce diffusion limitations, whereas micropores contribute to charge accumulation. This hierarchical porosity is especially important for the deep eutectic solvent (DES) electrolyte used in this study, where higher viscosity and larger effective ion sizes can limit accessibility to narrow pores. Therefore, the combination of accessible surface area and mesoporous transport pathways in the TiO_2_-modified materials contributes to the observed increase in capacitance and improved electrochemical response. The SEM and BET analyses have shown that TiO_2_ decoration increases the specific surface area and pore-size distribution of the carbon materials, providing more active sites for ion adsorption and facilitating faster ion transport during charge/discharge cycles [14]. The presence of TiO_2_ nanoparticles not only improves the overall conductivity of the carbon matrix but also introduces additional redox-active sites, as indicated by the sharp peaks in the XRD patterns, which contribute to the observed increase in capacitance.

The galvanostatic charge–discharge (GCD) results provide critical insights into the influence of light on the electrochemical performance of the carbon and TiO_2_-carbon composite materials. A clear trend emerges from the data, indicating that light significantly enhances charge storage and discharge times, as well as specific capacitance (C) in both samples.

Among the two carbon materials, PC exhibited the highest specific capacitance (15 ± 2 F g^−1^) and prolonged charge–discharge time under illumination. In the dark, however, PC’s discharge time and capacitance decreased slightly to 2.88 s (discharge) and 13 ± 2 F g^−1^, reflecting the absence of light-induced enhancements but retaining excellent cycling stability (92% retention at 1000 cycles).

The addition of TiO_2_ dramatically improved the charge storage properties of all composites, with PC+TiO_2_ achieving the highest capacitance (54 ± 5 F g^−1^) and prolonged discharge time (182.6 s) under illumination. This enhancement can be attributed to the pseudocapacitive properties of TiO_2_, which facilitate redox reactions and enhance charge storage under light. In the dark, the performance of PC+TiO_2_ diminished, with reduced discharge time (40.3 s) and specific capacitance (27 ± 1 F g^−1^). However, the retention of 98% capacitance after 1000 cycles in dark conditions demonstrates the stability and reliability of the composite, even in the absence of light.

For the BSG-based composite, light also played a significant role in boosting its electrochemical performance. BSG+TiO_2_ showed a marked reduction in capacitance from 49 ± 5 F g^−1^ (light) to 28 ± 3 F g^−1^ (dark), with corresponding reductions in charge–discharge times, further indicating the reliance of these systems on photo-induced charge carrier generation.

The carbon materials (PC and BSG) show only minor changes in electrochemical response under illumination, indicating that they do not exhibit significant intrinsic photoactivity. In contrast, the TiO_2_-containing composites display a clear light-induced enhancement, confirming that the semiconductor phase is primarily responsible for the observed photo-assisted behaviour. It is also important to note that TiO_2_ alone is not expected to exhibit significant capacitive performance under the present conditions due to its low intrinsic electronic conductivity, particularly in non-aqueous electrolytes. Therefore, the carbon matrix plays a critical role by providing a conductive network and accessible surface area, enabling efficient charge transport and allowing the photoactivity of TiO_2_ to contribute to the overall electrochemical response.

To further explore the impact of different electrolytes on discharge time and capacitance, the performance of aqueous and organic electrolytes, as well as an aqueous ChCl solution at the same molar concentration as used in the eutectic solvent, was evaluated. The GCD curves for these electrolytes are presented in Figures S9 and S10. These experiments revealed notable differences in electrochemical behaviour, highlighting the influence of the electrolyte medium on charge storage properties.

The GCD curves in Figure S7 compare the performance of the carbon-based composites in aqueous and organic electrolytes. Aqueous systems generally exhibited faster charge–discharge times and lower specific capacitances than their organic electrolyte counterparts. This discrepancy can be attributed to differences in ion dynamics and accessibility between aqueous and organic media, as well as to the influence of ion size and mobility on charge storage mechanisms.

Figure S8 demonstrates the performance of the aqueous ChCl solution compared to its eutectic counterpart. Notably, the ChCl aqueous solution exhibited shorter discharge times and lower specific capacitance than the eutectic system, emphasising the unique role of DES in enhancing electrochemical properties. The eutectic solvent likely provides a more favourable environment for ion transport and interaction with the carbon electrode surfaces, thereby enhancing capacitance and discharge behaviour.

These observations underline the critical role of the electrolyte composition in tailoring the electrochemical performance of carbon-based and TiO_2_-carbon composites. The comparison between aqueous and eutectic systems further demonstrates the advantages of DES, including higher capacitance and prolonged discharge times, making it a promising choice for advanced energy storage applications.

Overall, the results demonstrate that light significantly enhances the electrochemical properties of TiO_2_-decorated composites, primarily by influencing TiO_2_’s pseudocapacitive behaviour. While performance decreases in dark conditions, all samples retained substantial specific capacitance and excellent cycling stability, highlighting the robustness of these materials for energy storage applications in both illuminated and dark environments. This dual-mode functionality underscores the potential of these composites for versatile applications in next-generation supercapacitor systems.

The enhanced discharge time observed under illumination during GCD testing is attributed to the photogeneration of electron-hole pairs in TiO_2_ nanoparticles. The photogenerated electrons migrate into the conductive carbon matrix and are stabilised, leading to additional charge storage. During discharge, these photo-injected carriers are released along with conventional capacitive charges, thereby increasing discharge capacity. Furthermore, ongoing photogeneration during discharge sustains a higher charge-release rate, explaining why the extracted charge exceeds the initially stored charge during charging. This photocharging phenomenon confirms the light-responsive nature of the TiO_2_-carbon composites.

Additional experiments were performed to decouple the influence of illumination during charging and discharging. Charging under illumination, followed by discharging in the dark, resulted in significantly higher extracted charges for PC (Figure S9) and BSG (Figure S10), confirming that photogenerated carriers are primarily stored during charging. In contrast, discharging under illumination after dark charging only marginally improved discharge times, indicating that illumination during discharge alone has a minimal effect. These findings support the mechanism of enhanced charge storage via photoactivation during charging.

The influence of the DES electrolyte is particularly relevant under illumination, as reduced ion mobility and a structured ionic environment favour the accumulation and stabilisation of photogenerated charges at the TiO_2_–carbon interface, thereby enhancing the light-assisted capacitive response.

#### 3.7.2. Electrochemical Impedance Spectroscopy Analysis

The samples with higher capacitance (PC and PC+TiO_2_) were studied by electrochemical impedance spectroscopy (EIS) over a frequency range of 20 kHz to 0.1 Hz at a fixed potential of 0.5 V vs. Ag. The different carbon/composite samples were coated on the bare GC electrode and tested using ethaline as the electrolyte. Both samples subjected to EIS analysis showed a similar impedance response, as illustrated in Figure S11.

EIS spectra in Figure S11 reveal a depressed semicircle in the high-frequency region, which appears as a straight line in the low-frequency region, suggesting linear diffusion of charged ions throughout the electrodes’ pores. This circuit is modelled as a constant-phase element (CPE) loop and a Warburg diffusion (WD) impedance in an equivalent circuit.

The Ohmic resistance for all the systems shows very similar results, as shown in Table S3, since the electrolyte is the same across all systems. Upon analysing the charge-transfer resistances (Rt), it is evident that the PC electrode exhibits a superior resistance value for the electrochemical reaction. This enhancement is consistent with the morphological characteristics observed in SEM and STEM analyses, in which the TiO_2_ particles were well distributed throughout the carbon matrix, ensuring efficient electron transport and reducing overall electrode impedance.

The CPE coefficient (Q) is also higher, indicating a more pronounced capacitive effect within the electrode system. This elevated Q value suggests an enhanced ion adsorption capability on the electrode’s surface. Fu et al. [45] introduced a porous carbon material derived from crab shell that exhibited a Nyquist plot comparable to that of a Warburg region. This included a semi-circle with a small diameter, indicative of a low interfacial Rt [46], a trait beneficial for capacitive performance. Furthermore, Cai et al. [47] presented a carbon-TiO_2_ microsupercapacitor with a Nyquist plot and Warburg region similar to those observed for the carbon/TiO_2_ microsupercapacitor, demonstrating excellent capacitive behaviour.

Examining the CPE exponential factor (α), which is a metric for the electrode’s surface roughness, we typically observe values between 0.5 and 1. Values approaching 1 suggest diminished roughness and a more uniform current distribution across the surface. Based on the α values from this study, the electrode composed of PC+TiO_2_ exhibits the most porous structure. This observation aligns well with our SEM and BET analyses. Moreover, the current distribution of PC electrodes appears more homogeneous, as their α values are closer to 1. Notably, the Warburg diffusion coefficient (WD), related to the electrolyte’s diffusion within the electrode material, is highest for the PC electrode. This implies that impedance, in this case, is less influenced by mass transfer than in the other materials examined.

A more detailed analysis of the EIS results provides insight into the interfacial charge-transfer and ion-transport processes governing the electrodes’ electrochemical behaviour. The high-frequency intercept corresponds to the solution resistance (R_e_), which remains relatively similar for all samples, indicating comparable electrolyte and contact contributions. The semicircle observed in the high-to low-frequency region is associated with the charge-transfer resistance (Rct), reflecting the kinetics of electron transfer at the electrode–electrolyte interface. The TiO_2_-containing composites exhibit variations in Rct compared to the pristine carbon materials, indicating that the incorporation of TiO_2_ modifies the interfacial charge-transfer processes. This behaviour is consistent with the introduction of pseudocapacitive contributions associated with the TiO_2_ phase and the formation of a TiO_2_–carbon interface. The constant phase element (Q) and its associated exponent (α) describe the non-ideal capacitive behaviour of the electrode, which is influenced by surface heterogeneity and pore structure. The values obtained suggest a predominantly capacitive response with slight deviations from ideality, consistent with the hierarchical porous structure of the materials. In the low-frequency region, the impedance response reflects diffusion-controlled processes associated with ion transport within the porous electrode structure. The Warburg-related parameter indicates that ion diffusion is influenced by both the pore architecture and the properties of the deep eutectic solvent, which has a higher viscosity than aqueous electrolytes.

The combination of improved surface area, enhanced pore structure, and reduced charge-transfer resistance highlights the synergistic effect of TiO_2_ on the electrochemical performance of these biowaste-derived carbon materials. This finding is consistent with our previous work using a similar method on glycogen-derived carbon [14] in which the inclusion of TiO_2_ also resulted in significantly enhanced electrochemical properties, further validating the effectiveness of this approach for developing high-performance supercapacitor electrodes.

#### 3.7.3. Origin of Charge Storage and Light-Assisted Effects

The enhanced electrochemical response observed upon TiO_2_ incorporation and under illumination must be interpreted in terms of surface-confined charge storage rather than bulk faradaic redox reactions. In the applied potential window (0–1 V vs. Ag), classical Ti^4+^/Ti^3+^ bulk redox transitions are thermodynamically unlikely, particularly in a viscous, non-aqueous DES electrolyte.

Instead, the capacitive enhancement is attributed to a combination of (i) electric double-layer capacitance originating from the high-surface-area carbon framework and (ii) pseudocapacitive contributions associated with surface and near-surface states of TiO_2_, including oxygen vacancies, defect-related electronic states, and reversible surface hydroxyl interactions. These processes do not require long-range ion diffusion or phase transformation and are therefore consistent with the quasi-rectangular CV profiles observed.

Under illumination, TiO_2_ generates electron–hole pairs that are rapidly separated at the TiO_2_–carbon interface. The photogenerated electrons are preferentially transferred to and delocalised within the conductive carbon framework, while the corresponding holes remain associated with TiO_2_ surface states or defect-related sites. This spatial separation suppresses rapid recombination and increases the lifetime of photogenerated charges. The carbon matrix, therefore, acts not only as an electron sink but also as a conductive transport pathway that facilitates interfacial charge accumulation and stabilisation. As a result, illumination promotes a higher density of stored surface charge, which is reflected in the increased anodic current response and prolonged discharge times observed for the TiO_2_-containing composites. In this sense, the light-enhanced behaviour is consistent with a photo-assisted interfacial charge-storage mechanism rather than with dominant bulk Ti^4+^/Ti^3+^ conversion within the applied potential window. This photo-assisted charge accumulation manifests as increased anodic current and prolonged discharge times without invoking irreversible chemical reactions.

The asymmetry observed in the galvanostatic charge–discharge profiles under illumination reflects the continuous generation and partial retention of photogenerated carriers during charging. During discharge, these carriers are released alongside conventional capacitive charges, leading to an apparent increase in extracted charge relative to the pure electrochemical charging process. Such behaviour is characteristic of photo-assisted capacitive systems and does not imply a violation of charge conservation, but rather the superposition of electrochemical and photo-induced charge storage mechanisms.

Comparison with recent state-of-the-art systems also helps contextualise the present results. Recent literature on photo-assisted supercapacitors reports a wide range of device designs, from TiO_2_/carbon-fibre photoelectrodes showing light-enhanced capacitance to more complex TiO_2_/polymer or TiO_2_/graphene-based systems with substantially higher capacitance values and integrated photo charging behaviour. Likewise, conventional carbon–TiO_2_ composites designed for supercapacitor applications in aqueous electrolytes often reach higher absolute capacitance than the values reported here. Therefore, the significance of the present work does not lie in outperforming the best reported photo-supercapacitors on a capacitance basis, but in demonstrating a marine-biowaste-derived TiO_2_@C platform processed in a deep eutectic solvent and evaluated under light and dark conditions in a non-aqueous DES electrolyte. In this sense, the present study provides mechanistic and sustainability-oriented insights rather than serving as a record-breaking benchmark [48,49,50,51,52].

It is important to emphasise that the specific capacitance values reported here are modest compared to state-of-the-art carbon-based supercapacitors operating in aqueous electrolytes. This performance gap arises from the intrinsic limitations imposed by the DES electrolyte, including higher viscosity, lower ionic conductivity, and larger ions, which limit charge-propagation kinetics. However, the primary objective of this study is not to outperform conventional systems but to demonstrate how sustainable biocarbons and green electrolytes can be combined with semiconductor photoactivity to enable light-responsive capacitive behaviour. While no explicit modelling or simulation was performed in this work, the proposed mechanism is supported by the combined experimental evidence. The enhanced response under illumination is observed predominantly in the TiO_2_-containing composites.

In contrast, the parent carbon materials show only minor variations, indicating that the photoresponsive contribution is primarily associated with the semiconductor phase. Taken together with the morphological, porosity, compositional, and electrochemical data, these results support a mechanism in which light absorption by TiO_2_ generates charge carriers that are partially separated and stabilised at the TiO_2_/carbon interface. At the same time, the conductive porous carbon framework facilitates charge transport and interfacial charge accumulation. In this sense, the mechanism proposed here should be understood as an experimentally supported interfacial model consistent with the observed behaviour under both dark and illuminated conditions, rather than as a fully resolved theoretical description.

## 4. Conclusions

This study successfully demonstrates the potential of fish waste-derived carbon-TiO_2_ composites for mechanistically informative and sustainability-oriented energy storage systems. The innovative DES-mediated synthesis process facilitated the uniform decoration of TiO_2_ nanoparticles, significantly improving the surface area, pore structure, and electrochemical properties of the carbon materials. The inclusion of TiO_2_ not only enhanced the specific capacitance and cycling stability but also introduced light-responsive properties, enabling dual-mode performance in illuminated and dark environments.

The PC+TiO_2_ composite exhibited outstanding performance, achieving a specific capacitance of 54 ± 3 F g^−1^ and exceptional cycling stability (91% retention after 10,000 cycles) in a half-cell setup, highlighting its practicality for scalable energy storage systems. BSG+TiO_2_, despite its lower initial performance, demonstrated promising results.

By repurposing marine biowaste into advanced energy storage materials, this research addresses environmental concerns and advances sustainable technology development. The dual-mode capability, scalability, and eco-friendly synthesis of these composites make them promising candidates for next-generation supercapacitor applications. Future work could further optimise biocarbon precursors and electrode configurations to expand the potential of these materials across diverse energy storage systems. The insights provided herein underscore the importance of carefully distinguishing surface-confined capacitive processes from bulk redox phenomena in photo-assisted supercapacitors and highlight the need for future studies combining operando spectroscopy and device-level optimisation. Further confirmation of the detailed charge-transfer pathway through theoretical modelling or operando photoelectrochemical techniques would be valuable in future studies.

## Figures and Tables

**Table 1:** Textural properties of the materials obtained from N_2_ adsorption–desorption analysis, including specific surface area (SBET), total pore volume (Vp), and average pore diameter (Dp).

BET Analysis						
1000 °C 1 h	S_BET_ (m^2^ g^−1^)	V_micro_ (cm^3^ g^−1^)	V_meso_ (cm^3^ g^−1^)	V_total_ (cm^3^ g^−1^)	D_p_ (Å)	REF
PC	85 ± 1	0.029 ± 0.004	0.009 ± 0.001	0.038 ± 0.011	8.5 ± 0.2	[10]
BSG	30 ± 1	0.011 ± 0.002	0.019 ± 0.002	0.031 ± 0.009	8.7 ± 0.2	[11]
PC+TiO_2_	412 ± 2	0.195 ± 0.011	0.112 ± 0.001	0.307 ± 0.012	33.1 ± 0.3	This Work
BSG+TiO_2_	356 ± 2	0.154 ± 0.009	0.081 ± 0.003	0.235 ± 0.015	29.4 ± 0.4	This Work

V_micro_: micropore volume; V_total_: total pore volume; V_meso_: mesopore volume; D_p_: particle diameter.

**Table 2:** Atomic percentage (at.%) composition of the elements presented in PC, PC+TiO_2_, BSG and BSG+TiO_2_, carbonised for 1 h at 1000 °C.

At %				
Element	PC [10]	PC+TiO_2_	BSG [11]	BSG+TiO_2_
C 1s	78.4	50.4	81.4	41.2
N 1s	2.1	1.9	2.2	3.1
O 1s	14.3	35.1	4.1	34.6
Ti 2p	-	12.6	-	21.1

**Table 3:** Specific Capacitance of PC and PC+TiO_2_, BSG and BSG+TiO_2_ (1 A g^−1^) in a 3-electrode system (half-cell setup).

Galvanostatic Charge–Discharge Analysis 3-Electrode System (Half-Cell Setup)				
Sample	C (F g^−1^)	%C Retention		Reference
Bare TiO_2_/TiO_2_ nanotube electrode	lower/conductivity-limited	Varies		[40]
AC/TiO_2_ nanotube hybrid (aqueous)	128.4	-		[41]
Mesoporous carbon/TiO_2_ (aqueous)	280	95%		[42]
PC	15 ± 2	92 @1000 cycles		[10]
PC (dark)	13 ± 2	92 @1000 cycles		This Work
PC (H_2_SO_4_ 1M)	7	64 @1000 cycles		
PC (KOH 6M)	5.8	62 @1000 cycles		
PC (DMC: EMC 60:40 in 1M NEt_3_MeBF_4_)	11	70 @1000 cycles		
PC (Aqueous ChCl)	14 ± 1	83 @1000 cycles		
BSG	7 ± 1	71 @1000 cycles		[11]
BSG (dark)	8 ± 1	72 @1000 cycles		This Work
PC+TiO_2_	54 ± 3	97 @1000 cycles	91 @10,000 cycles	This work
PC+TiO_2_ (dark)	27 ± 1	98 @1000 cycles	91 @10,000 cycles	
PC+TiO_2_ (H_2_SO_4_ 1M)	14	73 @1000 cycles	-	
PC+TiO_2_ (KOH 6M)	12	73 @1000 cycles	-	
PC+TiO_2_ (DMC: EMC 60:40 in 1M NEt_3_MeBF_4_)	15	75 @1000 cycles	-	
PC+TiO_2_ (Aqueous ChCl)	32 ± 4	81 @1000 cycles	-	
BSG+TiO_2_	49 ± 5	95 @1000 cycles	85 @10,000 cycles	This work
BSG+TiO_2_ (dark)	28 ± 3	91 @1000 cycles	80 @10,000 cycles	

## Data Availability

The original contributions presented in this study are included in the article/Supplementary Materials. Further inquiries can be directed to the corresponding author.

## Supplementary Materials

The following supporting information can be downloaded at https://www.mdpi.com/article/10.3390/nano16090538/s1, Figure S1: (a) Dispersion of carbon material on the eutectic mixture during TiO_2_ formation and (b) biocarbon—TiO_2_ composite (BSG-TiO_2_) after the washing process; Figure S2: Setup for the electrochemical studies with a 60 W LED light (ON) and dark (OFF) effect; Figure S3: (a) CVs as a function of scan-rate in the range of 5 to 300 mV s^−1^ of 10 mM Fc. First scans shown. (b) Peak potentials and separations for the Fc/Fc^+^ redox couple and current ratios (Ic/Ia). (c) Increased CV potential range. (d) Randles-Ševčík analysis for the oxidation and reduction peak currents; Figure S4: Electrochemical impedance spectroscopy (EIS) at different potentials (including the oxidation and reduction peaks of Fc/Fc^+^ couple); Figure S5: SEM images of different fish waste precursors carbonised for 1 h at 1000 °C, at ×50 and ×100 magnification. This publication is licenced under CC-BY-NC-ND 4.0); PC [3] (© 2023 by the authors. Licensee MDPI, Basel, Switzerland. This article is an open access article distributed under the terms and conditions of the Creative Commons Attribution (CC BY) licence); BSG [4] (© 2023 by the authors. Licensee MDPI, Basel, Switzerland. This article is an open access article distributed under the terms and conditions of the Creative Commons Attribution (CC BY) licence); Figure S6: X-ray diffraction (XRD) patterns for PC+TiO_2_ (a) and BSG+TiO_2_ (b); Figure S7: Galvanostatic charge–discharge (GCD) curves of carbon-based composites in aqueous and organic electrolytes at 30 °C (1 A g^−1^); Figure S8: Galvanostatic charge–discharge (GCD) curves of carbon-based composites in an aqueous ChCl solution at 30 °C (1 A g^−1^); Figure S9: Galvanostatic charge–discharge (GCD) curves of PC+TiO_2_ composites in an ethaline at 30 °C (1 A g^−1^), in a three-electrode system, considering different light/dark conditions; Figure S10: Galvanostatic charge–discharge (GCD) curves of BSG+TiO_2_ composites in an ethaline at 30 °C (1 A g^−1^), in a three-electrode system, considering different light/dark conditions; Figure S11: Impedance response, at a frequency range of 20 kHz−0.1 Hz, at a fixed potential of 0.5 V vs. Ag, in Nyquist format; Table S1: EIS parameters at different potentials; Table S2: At % (atomic percentage %) composition of the elements presented in PC, PC+TiO_2_, BSG and BSG+TiO_2_, carbonised for 1 h at 1000 °C; Table S3: Regression results of equivalent circuit model parameters and their ±s confidence intervals.
