# Supplementary material for: Light-Enhanced Electrochemical Performance of Fish Waste-Derived Carbon-TiO2 Composites for Sustainable Energy Storage Systems
Source: Nanomaterials (Basel). 2026 Apr 29;16(9):538. doi: 10.3390/nano16090538 (PMC13165156; doi:10.3390/nano16090538)
Supplement: Supplementary file 1 [file nanomaterials-16-00538-s001.zip › nanomaterials-4212904-supplementary.pdf]

Supporting Information

1. TiO_2_C material

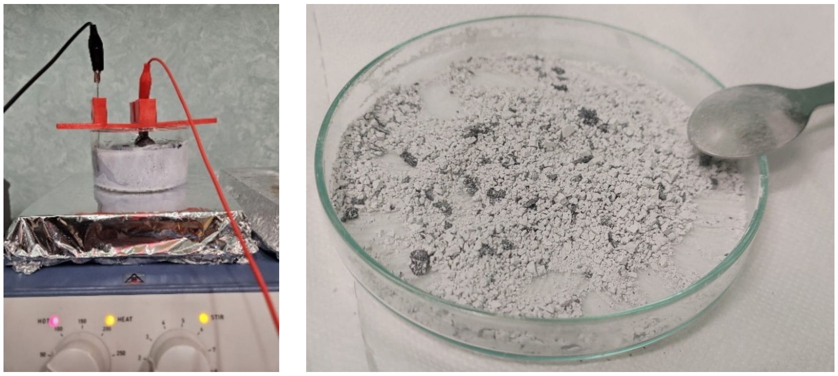

(a) (b)

Figure S1 (a) Dispersion of carbon material on the eutectic mixture during TiO_2 formation and (b) biocarbon – TiO_2 composite (BSG-TiO_2) after the washing process.

2. Light effect setup

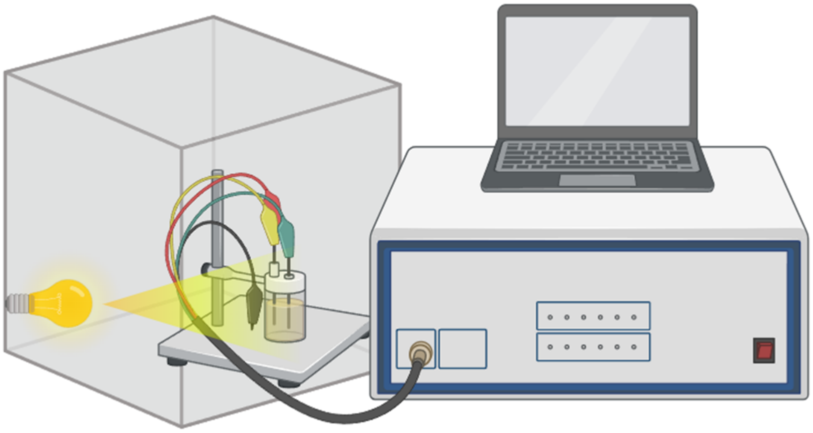

Figure S2 Setup for the electrochemical studies with a 60 W LED light (ON) and dark (OFF) effect.

3. Kinetics of the Fc/Fc$^+$ redox process

To establish the electrochemical window and interpret the nature of the charge storage in the TiO_2@C composites, it was carried out a detailed investigation using ferrocene (Fc) as a redox probe under identical experimental conditions. The Fc/Fc$^+$ couple, long considered a benchmark redox system in non-aqueous media, offers a reliable and reproducible reference point due to its single-electron, reversible redox chemistry and electrochemical stability within the potential range explored.

Figure S1a shows the voltametric behavior of 10 mM Fc/Fc$^+$ couple in ethaline on a glassy carbon electrode. A well-resolved oxidation/reduction pair is observed at ~0.63/0.54 V, respectively, yielding a peak separation that increases with scan rate (5–300 mV/s). The peak-to-peak separation (ΔEp) of the Fc/Fc$^+$ couple increases slightly with increasing

scan rate, evolving from ~80 mV at 5 mV/s to ~95 mV at 300 mV/s. The voltammograms exhibit the classical features of a diffusion-controlled redox process, including symmetric anodic and cathodic peaks with increasing peak currents as scan rate increases. This behavior is consistent with the findings of Armstrong et al. [2]. The high symmetry of the redox peaks and near-unity current ratio (Ia/Ic ≈ 1), as presented in **Figure S1b**, across scan rates further supports the electrochemical stability and reversibility of the Fc/Fc^+ system. Such behavior confirms the suitability of ferrocene as a redox standard in our system and affirms the reliability of our electrochemical potential window.

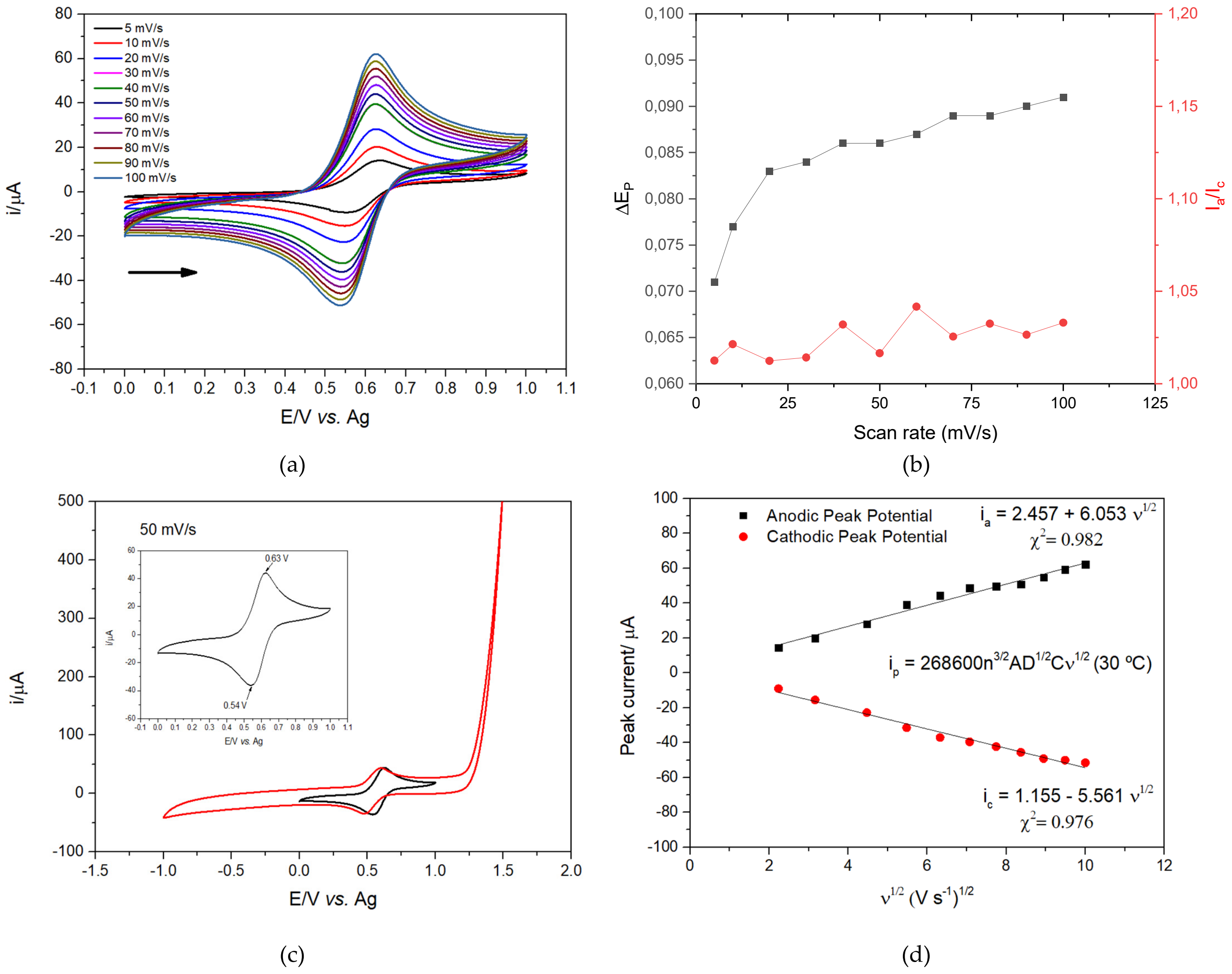

Figure S3 a) CVs as a function of scan-rate in the range of 5 to 300 mV s^{-1} of 10 mM Fc. First scans shown. b) Peak potentials and separations for the Fc/Fc^+ redox couple and current ratios (Ic/Ia). c) Increased CV potential range. d) Randles-Ševčík analysis for the oxidation and reduction peak currents.

Cyclic voltammetry over a broader window up to 1.5 V (**Figure S3c**) showed that the Fc^+ oxidation and reduction is consistent with the previously mentioned. This confirms that

the 0–1 V window employed in our material studies is within the electrolyte and redox reference's stable electrochemical regime. Finally, a Randles–Ševčík analysis was performed (**Figure S3d**), plotting peak current versus the square root of scan rate. The resulting linear relationship confirms the diffusion-controlled nature of the electron transfer, in line with ideal outer-sphere redox systems such as Fc/Fc^{+}. This behavior confirms the absence of adsorption or significant kinetic limitations over the potential range employed in the composite material studies.

To further evaluate the kinetics of the Fc/Fc^{+} redox process, a Randles–Ševčík analysis was performed by plotting the peak current (Ip) as a function of the square root of the scan rate ($v^{1/2}$). The resulting linear trend (**Figure S3d**) is consistent with diffusion-controlled electron transfer, as the Randles–Ševčík equation describes. The high linearity of the plot (R^2 > 0.98) confirms that no surface-adsorption or capacitive phenomena interfere with the redox behavior of ferrocene under the examined conditions. This indicates that the redox species are freely diffusing in solution and that mass transport, rather than charge transfer, dominates the response across the investigated scan rates. This result provides additional support for the use of Fc/Fc^{+} as a stable, well-behaved redox standard in our system. Further, it validates the potential window used to investigate pseudocapacitive behavior in the studied TiO_2@C materials.

This behavior indicates a quasi-reversible system, as further corroborated by Randles-Ševčík analysis reported in the literature. Importantly, the peak current ratio remained close to unity across scan rates, demonstrating the chemical and electrochemical stability of Fc and Fc^{+} under the experimental conditions. The Fc diffusion coefficient (D) is calculated using the following equation:

$$D = \left(\frac{\text{slope}}{268600\ n^{3/2}\ A\ C}\right)^2$$

n = 1 (for Fc/Fc^{+}, 1-electron transfer)

A = electrode area in cm^2 (0.0707 cm^2 for 3 mm diameter GC electrode)

C = concentration in mol/cm^3 (e.g., 10 mM = 1×10^{-5} mol/cm^3)

D_a =1.02 x 10^{-9} $cm^2\ s^{-1}$

D_c =8.58 x 10^{-10} $cm^2\ s^{-1}$

The diffusion coefficients determined from the anodic and cathodic slopes are similar, confirming the symmetric and reversible nature of the electron transfer process. Specifically, the anodic process yielded a diffusion coefficient of D_a = 1.02 x 10^{-9} $cm^2\ s^{-1}$, while the cathodic process yielded D_c =8.58 x 10^{-10} $cm^2\ s^{-1}$. The close agreement between these values further supports that the Fc/Fc^{+} couple exhibits fast electron transfer kinetics with minimal kinetic asymmetry under the studied conditions. Bahadori et al [3] presented a study for the determination of D at different concentrations of Fc in ethaline (at 25 ºC), presenting values ~3.08 x 10^{-8}, higher than the values obtained in this study, that can be explained by the not identical conditions of the measurements (temperature, DES viscosity and conductivity…). This high level of consistency strengthens the

validity of ferrocene as a reference system to anchor the potential window employed in the electrochemical characterization of the TiO_2@C composites.

Electrochemical impedance spectroscopy (EIS) provided further insights into the interfacial behavior of Fc across key potentials, as presented in **Figure S4**.

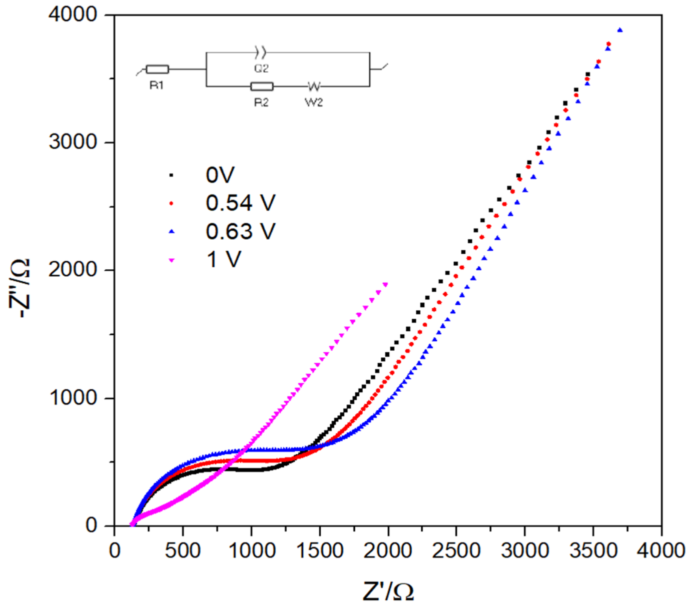

Figure S4 Electrochemical impedance spectroscopy (EIS) at different potentials (including the oxidation and reduction peaks of Fc/Fc^+ couple).

The results are summarized below (Table S1)

Table S1 EIS parameters at different potentials.

	**0 V**	**0.54 V**	**0.63 V**	**1 V**
**R1 (Ω)**	132.7	131.4	99.66	85.14
**Q2 (μF sa-1)**	6.09	6.52	7.12	11.25
**a**	0.897	0.881	0.891	0.914
**R2 (Ω)**	798.6	1018	1214	1104

As the potential increased toward 1 V, the series resistance (R_1) decreased significantly, while Q_2, indicative of the capacitive element associated with double-layer and pseudocapacitive processes, increased. The increase in Q_2 is accompanied by a high a-value (>0.9), suggesting a low degree of surface roughness and homogeneous current distribution. These findings strongly support the presence of enhanced faradaic processes at the upper part of the voltage window.

This behavior complements the work findings from the TiO_2@C materials, where enhanced capacitance was observed in the same potential range. The contribution from pseudocapacitive mechanisms is likely driven by the redox activity of the TiO_2 nanoparticles, which are known to be active in this range, as well as from heteroatom-doped carbon functional groups derived from the biomass precursor.

By benchmarking against the Fc/Fc^+ couple, it can be confirmed that the observed behavior is intrinsic to the composite structure and not an artifact of electrolyte

instability or experimental setup. These results validate the studied electrochemical protocol and reinforce the assignment of pseudocapacitance in the TiO_2-containing composites.

4. SEM analysis of Carbon materials

PC [3] BSG [4]

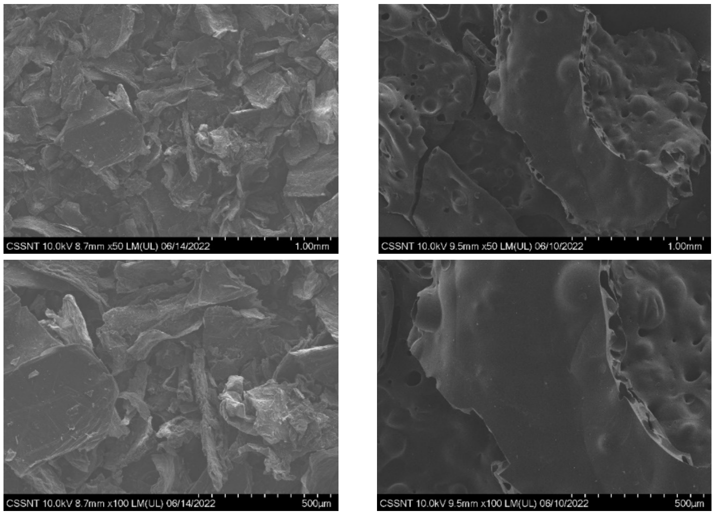

Figure S5 SEM images of different fish waste precursors carbonized for 1 h at 1000 °C, at ×50 and ×100 magnification. This publication is licensed under CC-BY-NC-ND 4.0); PC [3] (© 2023 by the authors. Licensee MDPI, Basel, Switzerland. This article is an open access article distributed under the terms and conditions of the Creative Commons Attribution (CC BY) license); BSG [4] (© 2023 by the authors. Licensee MDPI, Basel, Switzerland. This article is an open access article distributed under the terms and conditions of the Creative Commons Attribution (CC BY) license).

Table S2 At % (atomic percentage %) composition of the elements presented in PC, PC + TiO_2, BSG and BSG + TiO_2, carbonized for 1h at 1000 ºC.

	**At %**			
Element	**PC** [4]	**PC + TiO_2**	**BSG** [5]	**BSG + TiO_2**
C 1s	78.4	50.4	81.4	41.2
N 1s	2.1	1.9	2.2	3.1
O 1s	14.3	35.1	4.1	34.6
Ti 2p	-	12.6	-	21.1

5. XRD Analysis

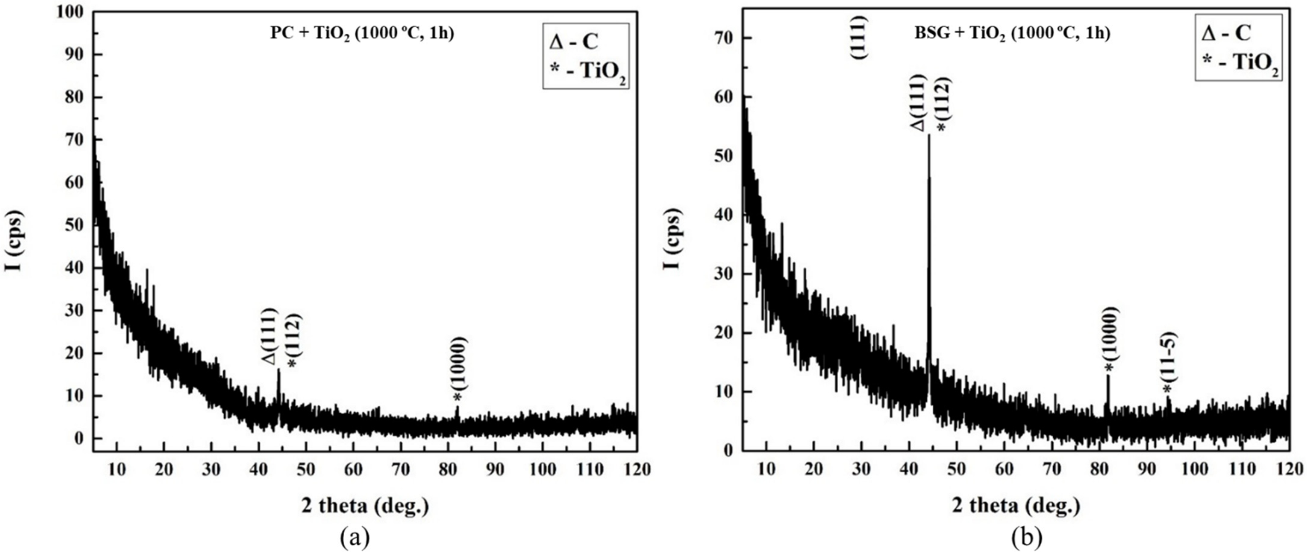

Figure S6 X-ray diffraction (XRD) patterns for PC+TiO_2 (a) and BSG+TiO_2 (b).

6. Electrochemical Analysis with aqueous and organic electrolytes

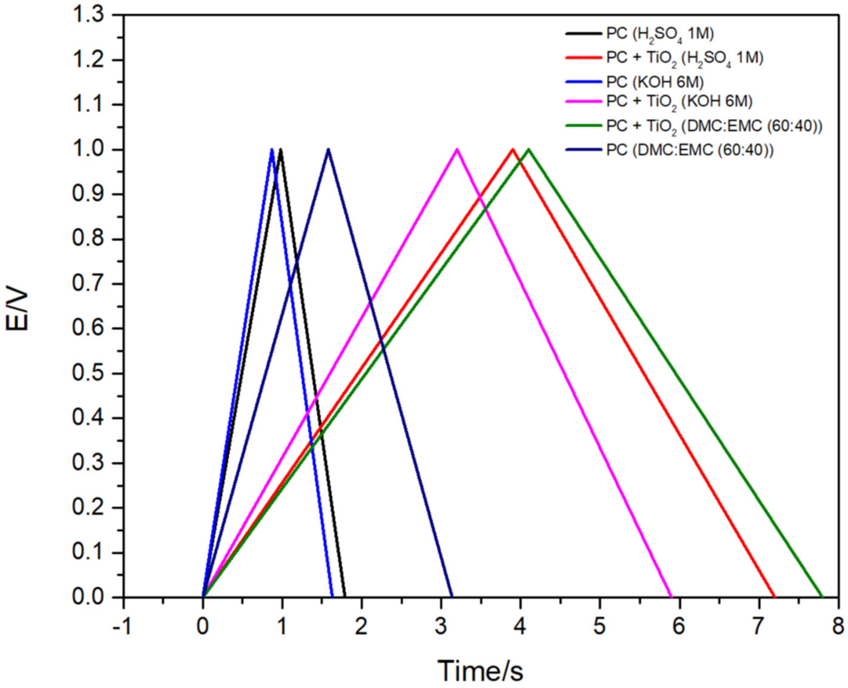

Figure S7 Galvanostatic charge-discharge (GCD) curves of carbon-based composites in aqueous and organic electrolytes at 30 °C (1 A g^{-1})

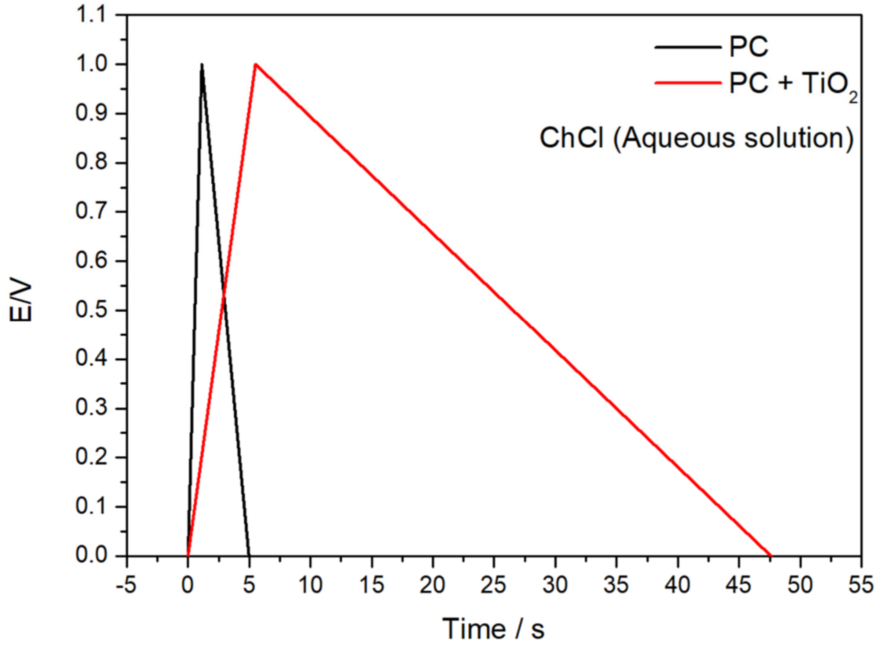

Figure S8 Galvanostatic charge-discharge (GCD) curves of carbon-based composites in an aqueous ChCl solution at 30 °C (1 A g^{-1})

7. GCD curves from these light/dark-separated experiments

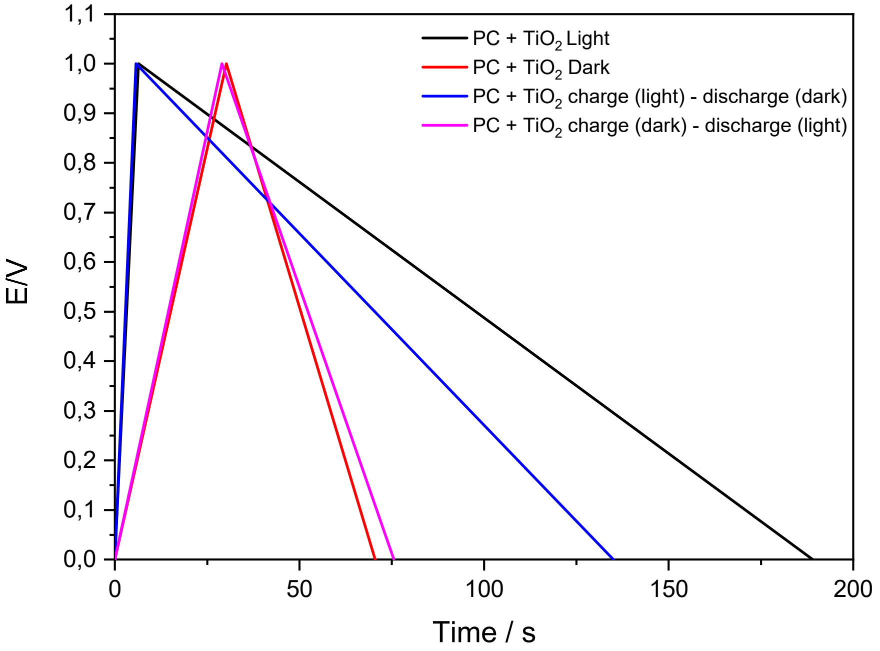

Figure S9 Galvanostatic charge-discharge (GCD) curves of PC + TiO_2 composites in an ethaline at 30 °C (1 A g^{-1}), in a three-electrode system, considering different light/dark conditions.

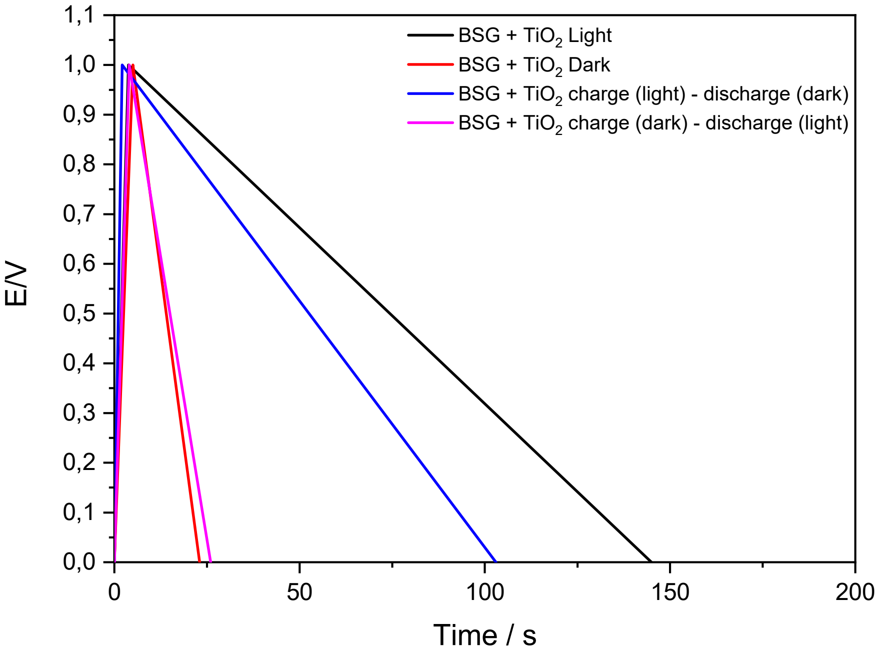

Figure S10 Galvanostatic charge-discharge (GCD) curves of BSG + TiO_2 composites in an ethaline at 30 °C (1 A g^{-1}), in a three-electrode system, considering different light/dark conditions.

8. Electrochemical Impedance Spectroscopy Analysis

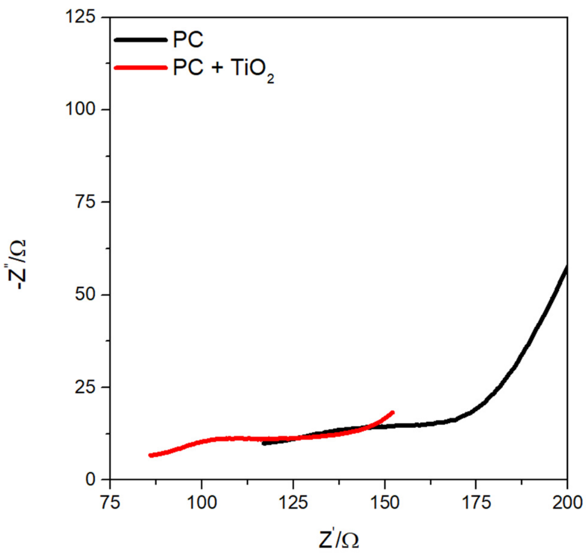

Figure S11 Impedance response, at a frequency range of 20 kHz−0.1 Hz, at a fixed potential of 0.5 V vs. Ag, in Nyquist format.

Table S3 Regression results of equivalent circuit model parameters and their ±s confidence intervals.

	R_e / Ω	R_t / Ω	α	Q/ μF $s^{\alpha-1}$	W_d/ Ω^{-1} $s^{0.5}$
PC	149.1 ± 0.5	73 ± 1	0.8 ± 0.1	156 ± 8	246 ± 45
PC + TiO_2	141.4 ± 0.7	84 ± 5	0.70 ± 0.05	144 ± 10	209 ± 5

9. References

[1] C.G. Armstrong, R.W. Hogue, K.E. Toghill, Characterisation of the Ferrocene/Ferrocenium Ion Redox Couple as a Model Chemistry for Non-Aqueous Redox Flow Batteries Research, n.d.

[2] L. Bahadori, N.S. Abdul Manan, M.H. Chakrabarti, Mohd.A. Hashim, F.S. Mjalli, I.M. AlNashef, Mohd.A. Hussain, C.T.J. Low, The electrochemical behaviour of ferrocene in deep eutectic solvents based on quaternary ammonium and phosphonium salts, Physical Chemistry Chemical Physics 15 (2013) 1707–1714. https://doi.org/10.1039/C2CP43077K.

[3] A.T.S.C. Brandão, R. Costa, S. State, P. Potorac, C. Dias, J.A. Vázquez, J. Valcarcel, A.F. Silva, M. Enachescu, C.M. Pereira, Chitins from Seafood Waste as Sustainable Porous Carbon Precursors for the Development of Eco-Friendly Supercapacitors, Materials 16 (2023). https://doi.org/10.3390/ma16062332.

[4] A.T.S.C. Brandão, S. State, R. Costa, L.-B. Enache, P. Potorac, J.A. Vázquez, J. Valcarcel, A.F. Silva, M. Enachescu, C.M. Pereira, Porous Carbon Materials Based on Blue Shark Waste for Application in High-Performance Energy Storage Devices, Applied Sciences 13 (2023). https://doi.org/10.3390/app13158676.
